# Optogenetic Control of Spine-Head JNK Reveals a Role in Dendritic Spine Regression

**DOI:** 10.1523/ENEURO.0303-19.2019

**Published:** 2020-02-12

**Authors:** Patrik Hollos, Jismi M. John, Jukka V. Lehtonen, Eleanor T. Coffey

**Affiliations:** 1 Turku Bioscience, University of Turku and Åbo Akademi University, Turku FI-20500, Finland; 2 Biochemistry, Faculty of Science and Engineering, Åbo Akademi University, Åbo FI-20500, Finland

**Keywords:** elimination, hippocampal neurons, kinase, optogenetics, spine, stress

## Abstract

In this study, we use an optogenetic inhibitor of c-Jun NH_2_-terminal kinase (JNK) in dendritic spine sub-compartments of rat hippocampal neurons. We show that JNK inhibition exerts rapid (within seconds) reorganization of actin in the spine-head. Using real-time Förster resonance energy transfer (FRET) to measure JNK activity, we find that either excitotoxic insult (NMDA) or endocrine stress (corticosterone), activate spine-head JNK causing internalization of AMPARs and spine retraction. Both events are prevented upon optogenetic inhibition of JNK, and rescued by JNK inhibition even 2 h after insult. Moreover, we identify that the fast-acting anti-depressant ketamine reduces JNK activity in hippocampal neurons suggesting that JNK inhibition may be a downstream mediator of its anti-depressant effect. In conclusion, we show that JNK activation plays a role in triggering spine elimination by NMDA or corticosterone stress, whereas inhibition of JNK facilitates regrowth of spines even in the continued presence of glucocorticoid. This identifies that JNK acts locally in the spine-head to promote AMPAR internalization and spine shrinkage following stress, and reveals a protective function for JNK inhibition in preventing spine regression.

## Abstract (precis)

In this study, we use an optogenetic inhibitor of c-Jun NH_2_-terminal kinase (JNK) in dendritic spine sub-compartments of rat hippocampal neurons. We show that JNK inhibition exerts rapid (within seconds) reorganization of actin in the spine-head.

## Significance Statement

Identifying mechanisms that underlie dendritic spine elimination is important if we are to understand maladaptive changes that contribute to psychiatric disease. Compartment-specific, fast-acting tools can expedite this endeavor. Here we use a light-activated inhibitor of c-Jun NH_2_-terminal kinase (JNK) to control kinase activity specifically in dendritic spines. Light-activation of the JNK inhibitor reduces AMPA receptor removal and spine regression in response to corticosterone and NMDA stress. Furthermore, we find that the anti-depressant drug ketamine lowers JNK activity in hippocampal neurons and prevents spine regression, though direct JNK inhibition is more effective. This study identifies a role for JNK in spine regression and may be relevant for endocrine control of synaptic strength and for conditions where chronic glucocorticoid stress leads to spine elimination.

## Introduction

Dendritic spines account for >90% of glutamatergic synapses in the brain and are essential sites for processing electrochemical input (Nimchinsky et al., 2002). New spine growth and synapse formation occur throughout life and stabilization of synapses correlates with memory formation and consolidation (Matsuzaki et al., 2001 ; Noguchi et al., 2011; Attardo et al., 2015; Berry and Nedivi, 2017). Turnover of spines also effects learning and memory performance and transient spines permit Hebbian-like plasticity (Holtmaat and Caroni, 2016). On the other hand, excessive spine elimination is a hallmark of schizophrenia and major depressive disorder (Musazzi et al., 2011; Duman and Duman, 2015; Varidaki et al., 2016; Wohleb et al., 2018), and postmortem studies report that there are spine deficits in prefrontal cortex and hippocampus of these individuals (Michelsen et al., 2007; Duman, 2009; Hajszan et al., 2009; Duman et al., 2012; Duman and Aghajanian, 2012; Radley et al., 2013). In addition to this, classical anti-depressant drugs such as fluoxetine and desipramine have been shown to reverse spine loss (Hajszan et al., 2009; Chen et al., 2010; Rubio et al., 2013; McAvoy et al., 2015), suggesting that spine loss may contribute to the underlying pathology. Even the fast-acting anti-depressant ketamine has been shown to prevent net spine loss in a rodent chronic stress model, leading to recovery of dysfunctional circuit activity (Moda-Sava et al., 2019; Treccani et al., 2019). Thus, spine regression while a fundamental feature of normal synaptic plasticity, is also associated with psychiatric disorders. Molecular drivers of spine shrinkage are largely unknown.

c-Jun NH_2_-terminal kinases (JNKs) exert pleiotropic functions during development of the nervous system, regulating cell death and proliferation as well as migration and dendrite arborization (Weston and Davis, 2007; Westerlund et al., 2011; Björkblom et al., 2012; Coffey, 2014; Komulainen et al., 2014). However, JNKs are first and foremost recognized as stress-activated protein kinases that are strongly activated by a variety of cellular stresses including cytokines and glucocorticoids (Qi et al., 2005; Kyriakis and Avruch, 2012), and stress-activated JNK is associated with synaptic deficits in mouse models of Alzheimer’s disease (Sclip et al., 2014). Moreover, activation of JNK2 and JNK3 in hippocampus was shown to be necessary for stress-induced learning deficit in mice (Sherrin et al., 2010). It has also been shown that interleukin, β-amyloid and restraint-stress-induced activation of JNK impairs hippocampal LTP (Curran et al., 2003; Costello and Herron, 2004; Sherrin et al., 2011). Evidence from animal studies and human genetics studies suggest that dysregulation of the JNK pathway may be linked to psychiatric disorders (Winchester et al., 2012; Kunde et al., 2013; McGuire et al., 2017; Mohammad et al., 2018; Kowalchuk et al., 2019; Openshaw et al., 2019). For example, inhibition or genetic deletion of *Jnk1* reduces anxiety and depressive-like behaviors in mice (Mohammad et al., 2018), and MAP2K7 heterozygote mice display brain imaging endophenotypes and behaviors related to schizophrenia (Openshaw et al., 2020). Although JNK has been shown to regulate synaptic plasticity in learning and the JNK pathway is genetically associated with disorders of synaptic function; mechanistic study of JNK function in dendritic spines has been limited due to lack of tools that allow spatiotemporal control of the kinase solely in the spine-head.

Here, we exploit an optogenetic inhibitor of JNK to control kinase activity in spines. This reveals that stress activated JNK triggers AMPA receptor internalization and rapid spine retraction following activation by NMDA or corticosterone. The antidepressant drug ketamine, suppresses activation of JNK and helps prevent spine loss; however, direct JNK inhibition elicits a faster and more potent block of receptor internalization and spine retraction and reduces spine elimination even when administered 2 h after glucocorticoid stress. These results indicate that JNK drives dendritic spine regression in response to stress.

## Materials and Methods

### Plasmid construction

Rat β-actin was obtained by PCR and was ligated to the EcoRI site of the pVenus vector followed by exchanging the Venus tag for mCherry using NheI/BsRGI sites. NES-c-Jun(1-146) was prepared by PCR-based methods from pcDNA3-mJIP1a (Flag-JBD; gift from Martin Dickens, Leicester) and cloned into eGFP-C1 (Clontech). Subsequently, GFP was replaced for mCherry using NheI/BsrGI sites to yield mCherry-NES-c-Jun(1-146). The photoactivatable pLuc-LOV2WTJαWT-JBD (*LOV2-JBD*), the *lit-state* mutant pLuc-LOV2WTJαIE-JBD and the *dark-state* mutant pLuc-LOV2C450AJαWT-JBD, were prepared by ligating JNK interacting protein-1 (JIP1 144-154) “RPKRPTTLNLF” downstream of LOV2 to generate a specific inhibitor of JNK, as previously described (Melero-Fernandez de Mera et al., 2017). LOV2 was generated by gene synthesis from *Avena sativa* LOV2 with codon optimization. GFP-LOV2WTJαWT (LOV2-JBD) was prepared by PCR insertion of wild-type LOV2 into the pEGFP-C1 vector with overhanging SalI/SacII sites. To generate a red-shifted Förster resonance energy transfer (FRET) sensor that would not overlap with the LOV2 absorption peak, the JNKAR1EV probe (provided by Michiyuki Matsuda, Kyoto University) was modified by inserting mRuby2 and Clover tags (gifts from Michael Lin, Addgene plasmids #40260 and #40259, respectively), in place of ECFP and YPET using EcoRI/XhoI and NotI/SalI sites, respectively. pCI-SEP GluR2 (SEP-GluR2) was a gift from Robert Malinow (Addgene plasmid # 24001) and eYFP-C1 was from Clontech.

### Structure prediction

To generate an estimated view of possible 3D conformations of the LOV2-JBD tools in *dark-state* and *lit-state*, we used MODELLER (Sali and Blundell, 1993). This software uses known Protein Data Bank-derived structures as templates for prediction of least constrained conformations and is reported to generate chemically correct models (Wallner and Elofsson, 2005). PDB ID 2VOU and 2V1A cryo-trapped wild-type and *dark-state* structures of LOV2 (Halavaty and Moffat, 2007) were used as templates to predict the dark-state after alignment with our *dark-state* mutant. PDB ID 2V0W and 2V1B were used as templates for the *lit-state* mutant (Heo et al., 2004). Energy minimization used Yet another Scientific Artificial Reality Application (YASARA v16; Krieger et al., 2009). C*α* atoms were aligned with templates to obtain root mean squared deviation (RMSD) values for the models. The top hit energy minimized model from MODELLER v9.11 was used.

### Immunocytochemistry and wide field imaging

Phosphorylated c-Jun (p-Jun) was detected using (1:200) anti-phospho-c-Jun Ser 63 II (#9261) from Cell Signaling Technology, which has been shown to be specific (Brecht et al., 2005) and detected with anti-rabbit Alexa Fluor 488, 568, or 405 (1:500) as indicated, from Invitrogen Corporation. Hoechst-33342 and Mowiol mounting media were from Invitrogen. For immunostaining, neurons grown on 13 mm coverslips were washed once with 1-ml ice-cold PBS and fixed using 4% PFA for 30 min. After 3 × 1 ml washes with PBS, cells were permeabilized using 1% Triton X-100 in PBS for 3 min and washed again (3 × 1 ml) with PBS. Non-specific binding was blocked using 10% fetal bovine serum for 1 h. Primary and secondary antibodies were added consecutively, each for 1-h incubations. Coverslips were mounted in 8-μl Mowiol containing DABCO anti-fade and analyzed using a Zeiss LSM-780 with appropriate laser illumination. Imaging of p-c-Jun and mCherry-NES-Jun was conducted with a Leica DMRE upright microscope and 40× air objective. Identical acquisition parameters were used for all cells so that the images were quantitatively comparable. Line intensities (15 μm) were measured from the dendritic shaft using ImageJ (NIH). Cytoplasmic JNK activity was calculated from non-saturated line intensity ratios: [intensity p-Jun]/[intensity mCherry-NES-Jun]. Ratiometric images were generated using the Ratio Plus plugin for ImageJ.

### Hippocampal neuron isolation and maintenance

Newborn Sprague Dawley rats of either sex were decapitated and the hippocampus rapidly removed into dissection media [1 M Na_2_SO_4_, 0.5 M K_2_SO_4_, 1 M MgCl_2_, 100 mM CaCl_2_, 1 M HEPES (pH 7.4), 2.5 M glucose, and 0.5% phenol red]. Meninges were removed and tissue pieces collected into dissection media containing 10% KyMg, followed by washing. Tissues were incubated with 10 U/ml papain (Worthington, 3119) for 15 min at 37°C, repeated two times. Papain was inactivated by incubation with 10 mg/ml trypsin inhibitor (Sigma, T9128) for 2 × 5 min at 37°C. Tissue was then dissociated by trituration to yield a homogenous solution of cells. Cultures were maintained in Neurobasal-A (Thermo Fisher Scientific), supplemented with 2 mM glutamine, 50 U/ml penicillin, 50 μM streptomycin, and B27 Neuronal supplement (Gibco, Thermo Fisher Scientific). Cells were transfected with a DNA mix comprising 30% of transgene vector and 70% of pCMV vector lacking transgene, to give a total of 0.5 μg DNA per 24-well plate well, using 7–10 d *in vitro* (DIV) cells using Lipofectamine 2000 (Thermo Fisher) following the manufacturer’s instructions. Experiments were conducted in hippocampal pyramidal cells at 16–18 DIV.

### Photoactivation of LOV2 variants

**Figure 1.**
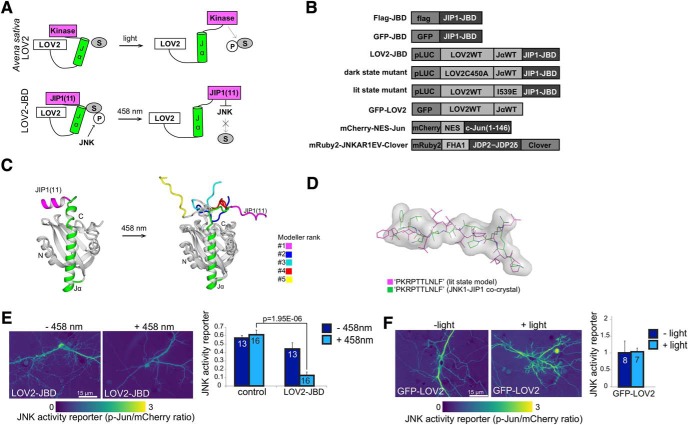
Design and validation of the LOV2-JBD inhibitor. ***A***, Schematic showing activation of LOV2 photo-domain from *Avena sativa* by light. The proposed mode of action of LOV2-JBD is shown in the lower panel where an 11-mer peptide inhibitor of JNK is released from the constrained conformation on photostimulation, facilitating binding to and inhibition of JNK. ***B***, Constructs used in this study. ***C***, Superimposed, top five *lit-state* model predictions for LOV2-JBD. In the top-ranked model, JIP1(11) (magenta) takes on a relaxed conformation projecting away from the core. ***D***, JIP1(11) from the *lit-state* model (magenta) superimposed on the crystal structure of JIP1(11) from the JNK1-JIP1 co-crystal (green). ***E***, mCherry-NES-Jun fluorescence provides a surrogate reporter of JNK activity in hippocampal neurons expressing mCherry-NES-Jun (control) or mCherry-NES-Jun with LOV2-JBD. Reporter activity is fluorescence intensity of phosphorylated c-Jun (P-Jun)/mCherry fluorescence intensity. Photostimulation of LOV2-JBD reduces JNK activity in hippocampal neurons; however (***F***) photostimulation of GFP-LOV2 does not. Data on *dark-state* and *lit-state* mutants are in Extended Data Figure 1-1. Mean data ± SEM and Student’s *t* test *p* values are shown. Cell numbers (from at least two experimental repeats) are indicated on the bars.

Cells for live cell imaging were plated on glass bottom dishes (Greiner Bio-One GmbH) or in black walled glass bottom dishes (PELCO), when light sensitive constructs were used. Cells transfected with photosensitive constructs were protected from ambient light in a foil-covered dark box for 5 min before imaging. Handling was performed in a darkened room where the computer monitor was set on minimum light level for set-up and switched off during experiments. In experiments where photoactivation was used and subsequently monitored, we used a Zeiss LSM-780 or Zeiss LSM-880 with Airyscan microscope, as indicated. This was equipped with an incubator chamber: 37°C, 5% CO_2_, and 10× objective was used with a 458-nm argon laser, scan speed of 1.68 μs/pixel in “live scan” mode for 30 s, followed by immediate fixation with 4% PFA; 3% laser power (0.4-mW irradiance) was used for LOV2 illumination, unless otherwise indicated. Regions of interest (ROIs) in dendritic spines were illuminated with the 458-nm laser for 1 s with 1.68 μs/pixel dwell speed using 63 × 1.2 W objective. For Figure 1*F*, where field illumination was used, hippocampal neurons in a 24-well plate were illuminated by placing 40 cm below a fluorescent lamp (Phillips, PL-2 11W/865/2P ICT/25) for 30 s followed by immediate fixation of cells with 4% PFA.

10.1523/ENEURO.0303-19.2019.f1-1 Extended Data Figure 1-1 Testing functionality of LOV2-JBD tools in neurons expressing mCherry-NES-Jun as a JNK activity reporter. ***A***, Fluorescent micrographs from 16-d hippocampal neurons expressing the mCherry-NES-Jun reporter in the presence or absence (control) of the *dark-state* and *lit-state* mutants of LOV2-JBD as indicated. Micrographs show ratio images of phospho-Ser63-c-Jun (P-Jun)/mCherry-NES-Jun (mCherry) fluorescence. Scale bar = 15 μm. ***B***, JNK activity (from multiple experiments as shown in ***A***) is the ratio of phosphorylated c-Jun normalized to mCherry-NES-Jun reporter expression. The “*lit-state*” mutant of LOV2-JBD reduced JNK activity even without photostimulation. The “*dark-state*” mutant did not significantly alter JNK activity even in the presence of light. Mean data ± SEM are shown; *p* values are shown from repeated measures one-way ANOVA and are indicated above the histogram bars. Total number of cells analyzed from at least two experimental repeats are indicated on the bars. Download Figure 1-1, TIF file.

### FRET imaging and analysis

FRET analysis was performed using the Zeiss FRET module. Control (acceptor or donor alone) and FRET images were acquired by using the Lambda stack scan mode on the Zeiss-LSM 780 to achieve appropriate spectral separation. For mRuby2-JNKAR1EV-Clover FRET imaging, the donor (Clover) was excited using 0.4 mW of a 488 nm laser and a 63× objective. mRuby2 was excited using 0.24 mW of a 543 nm laser. LOV2 was excited using 0.4 mW of a 458 nm laser. To calculate FRET efficiency, several ROIs were drawn on dendrites, and Youvan’s method was used to calculate the FRET response as follows: Fc = (fret_gv_ – bg_fret_) – cf_don_*(don_gv_ – bg_don_) – cf_acc_*(acc_gv_ – bg_acc_), where: *Fc* = FRET concentration, *gv* = gray value intensity, *bg* = background intensity, *don* = donor image, *acc* = acceptor image, *cf* = correction factor. To compare FRET efficiency between different cellular compartments, normalized FRET (N-FRET) values were calculated as following N-FRET: *Ff – ([don_corr_] – [acc_corr_])/√[(G(Fd)(Fa)]*, where *Ff* the FRET image in FRET channel, *don_corr_*= donor image with donor excitation, *acc_corr_*= acceptor image with acceptor excitation, *Fd* = emission cross talk of the donor in the FRET channel, *Fa* = emission cross talk of the acceptor in the FRET channel, *G* = donor emission factor in the donor channel due to FRET, relative to the acceptor emission due to FRET in the FRET channel.

### Measurement of actin dynamics in dendritic spines

**Figure 2.**
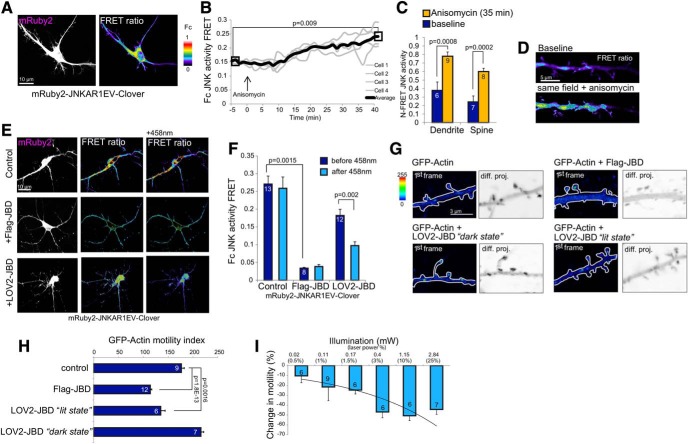
Photoactivation of LOV2-JBD reduces actin dynamics in dendritic spines. ***A***, Hippocampal neurons expressing mRuby2-JNKAR1EV-Clover FRET reporter provide a real-time readout of JNK activity. The mRuby2 image (left) indicates reporter expression and FRET image (right) indicates JNK activity, which is high in the cytoplasmic compartment. The look up table (LUT) shows FRET ratios (Fc) from 0 to 1. ***B***, mRuby2-JNKAR1EV-Clover FRET reporter activity from hippocampal neuron dendrites increases following JNK activation following anisomycin (10 μM) treatment. ***C***, N-FRET shows that JNK is activated in dendrites and spines 40 min following anisomycin (10 μM). Mean data ± SEM are shown; p values are from Student’s t test. Extended Data Figure 2-1 shows mRuby2-JNKAR1EV-Clover FRET has improved dynamic range compared to YPET-JNKAR1EV-CFP. ***D***, Representative image of mRuby2-JNKAR1EV-Clover FRET in dendritic spines. ***E***, FRET images from live cell analysis indicate Flag-JBD inhibits JNK, as does LOV2-JBD following photostimulation for 30 s with 0.4 mW of 458-nm laser. ***F***, Corrected FRET (Fc) from multiple experiments as described in ***E***. Measurements were from five regions of interest per cell, from four cells from at least two experimental repeats. Mean data ± SEM are shown. Adjusted p values are shown from repeated measures one-way ANOVA with Bonferroni correction. Extended Data Figure 2-2 shows independent readout of JNK activity changed due to excitation of the FRET probe with 488-nm laser. ***G***, GFP-actin displacement is shown in arithmetic difference projections (diff. proj.) depicting summed GFP-actin displacements from 11-min imaging. Darker pixels represent higher motility. Scale bar = 3 μm. ***H***, Calculated motility index from several recordings as described in ***G***. Flag-JBD and LOV2-JBD *lit-state* mutants significantly reduce spine motility. Mean data ± SEM are shown. Adjusted *p* values are shown from repeated measures one-way ANOVA with Bonferroni correction ***I***, The minimal illumination required to photoactivate LOV2-JBD is shown. Motility changes from neurons expressing mCherry-actin plus LOV2-JBD and exposed to increasing 458-nm illlumination are shown; 0.4 mW (achieved using 3% laser power) was the minimum irradiance needed to elicit a maximal response. Motility was calculated from four to six spines per cell and two to three cells per condition from at least two experimental repeats.

Hippocampal neurons expressing mCherry-actin (or GFP-actin as indicated) together with LOV2 variants (as indicated), were imaged using a Zeiss-LSM 780 (or LSM-880 in Airyscan mode where indicated) microscope and a 63× objective (C-Apochromat, 1.2 numerical aperture) for live cell imaging or with a Zeiss LSM-880 Airyscan microscope for live-cell 3D imaging of spines. A 543-nm laser at 3% power was used to image mCherry-actin, and a 488-nm laser at 6% power for GFP-actin (Fig. 2 only), in dendritic spines from secondary dendrites, located 20–30 μm from the soma were measured. The stack registration plugin from ImageJ was used to correct *x*, *y* drift from time-lapse series and images were then median filtered with 1-pixel radius to enhance the signal-to-noise ratio. Scan speed was 1.68 μs/pixel and the time between frames was 6 s. For fast 3D Zeiss LSM-880 Airyscan imaging, scan speed was ∼150 ms for every 0.22 μm *z*-slice. Photoactivation of LOV2-constructs was achieved using the 458-nm laser line at 3% laser power (0.4-mW).

10.1523/ENEURO.0303-19.2019.f2-1 Extended Data Figure 2-1 mRuby2/Clover pairing improves JNK FRET reporter sensitivity. ***A***, The sensitivity of FRET response was compared using the EYFP-JNKAR1EV-CFP FRET reporter and the newly generated mRuby2-JNKAR1EV-Clover reporter. Reporter activity was measured in 16-d hippocampal neurons treated with anisomycin (10 μM). ***B***, N-FRET was measured from multiple cells expressing both reporters. mRuby2-JNKAR1EV-Clover provided improved dynamic range compared to EYFP-JNKAR1EV-CFP (dendrite, *p* = 0.000167). Measurements were from five regions per cell and four cells per experiment on two separate experiments. Mean data ± SEM are shown. Download Figure 2-1, TIF file.

10.1523/ENEURO.0303-19.2019.f2-2 Extended Data Figure 2-2 The 488-nm irradiation in neurons expressing LOV2-JBD partially inhibits JNK. Fluorescent micrographs from 16-d hippocampal neurons expressing mCherry-NES-Jun+LOV2-JBD in the presence, or absence of 488-nm irradiation mimicking Clover/FRET channel excitation (0.4 mW). Micrographs show ratio images of phospho-Ser63-c-Jun (P-Jun)/mCherry-NES-Jun (mCherry) fluorescence. Scale bar = 15 μm. ***B***, JNK activity (from multiple experiments as shown in ***A***) is the ratio of phosphorylated c-Jun normalized to mCherry-NES-Jun reporter expression. Mean data ± SEM are shown, *p* value is obtained by Student’s *t* test. Download Figure 2-2, TIF file.

The motility of GFP, mCherry-actin or YFP within spines was quantified using arithmetic difference projection analysis as described by (Fischer et al., 1998; Björkblom et al., 2012). Displacement images were generated between consecutive frames of 6-s interval and the sum of all displacement is projected. These provided a 2D quantitative view of movement across the time frame where pixel intensity corresponds to actin displacement. Arithmetic projections were inverted for visualization. The “motility index” describes the average intensity in ROIs from arithmetic projections. The “motility ratio” was the ratio of motility indices obtained before and after light activation. To view the temporal detail of dynamic changes, RGB temporal color-coded difference projections were generated using frame-by-frame difference stacks. For this we used the ImageJ Hyperstack color-code plugin with customized LUT. A white, or Gaussian mix of colors indicates continuous motility. Each time frame is represented by a unique hue. For these fast motility measurements, LSM-880 Airyscan images were acquired in fast-scan mode, taking a 6 × 6 × 4 (*xyz*) μm voxel scan approximately every second.

### Measurement of synaptic GluR2 receptors and spine-head volume

To measure surface GluR2 levels at synapses, hippocampal neurons expressing SEP-GluR2 (which undergoes fluorescence quenching on internalization into endocytic vesicles) and mCherry-actin underwent time-lapse imaging for 40 min using the Zeiss LSM-880 in Airyscan mode using the 488-nm laser at 0.8% (0.19 mW) power so as to minimize inadvertent photoactivation of LOV2. Fluorescence intensity for each fluorescent protein was measured from circular ROIs in spines. To determine relative GluR2 surface expression, the ratio of SEP-GluR2 to mCherry-actin or YFP was calculated. To analyze spine head volume, ROI voxels were drawn encompassing the entire volume occupied by visible mCherry-actin or YFP in the spine head throughout the time lapse as previously (Basu et al., 2018; Hruska et al., 2018), ensuring that the entire 4D footprint was included in the analysis. The *z*-depth was 4 μm and *x*, *y* dimensions varied according to individual spine size. Spines were imaged in 3D with a *z*-section of 0.2-μm scan speed of three frames per second. Relative changes in dendritic spine volume were estimated from maximum intensity projections from these images. To calculate a baseline spine volume, maximum projections from 3 min of image acquisition before treatment were averaged. Subsequent recordings were normalized to this value. For fixed cell analysis, relative changes in dendritic spine volumes and SEP-GluR2 to mCherry-actin ratios were normalized to control conditions (before treatment). Boundaries of spine heads were drawn from maximum intensity images where the border was defined by change in signal:noise >10 compared to mean background.

### Hippocampal neuron pharmacological treatments

Anisomycin (Sigma) was added at 10 μM concentration in growth media to hippocampal neurons during live cell imaging after 5 min of baseline recording. To produce chemical long-term depression (LTD), hippocampal neurons at 16–18 DIV were treated with 20 μM NMDA (Tocris Biosciences) for 3 min in Neurobasal-A, after which conditioned growth medium was added back, as previously described (Lee et al., 1998). Corticosterone (100 nM; Sigma-Aldrich Finland Oy) was added to hippocampal neurons following 5 min of baseline recording or 2 h before or after ketamine treatment as indicated. Ketamine (10 μM; Tocris Biosciences) was added to hippocampal neurons 2 h before recording. Latrunculin B (1 μM; Sigma-Aldrich Finland Oy) or Phalloidin (500 nm; Sigma-Aldrich Finland Oy) was added to hippocampal neurons following 5 min of baseline recording.

### Statistical analysis

Student’s two-tailed *t* tests were used to calculate *p* values where two groups were compared. Repeated measures one-way ANOVA was used to compare multiple groups and *p* values were corrected by Bonferroni correction. Error bars represent SEM. In each case, measurements were taken from multiple cells and spines from separate experiments, and statistical analysis is detailed in the corresponding legend.

Animal procedures were performed in accordance with the Turku Central Animal Laboratory regulations and national guidelines.

## Results

We generated an optically controllable inhibitor of JNK to enable localized inhibition of JNK function exclusively in dendritic spines without altering JNK activity in other compartments. To do this, we replaced the kinase domain from *A. sativa* phototropin with an 11-mer peptide inhibitor of JNK (JNK binding domain; JBD), a highly specific peptide inhibitor of JNK which does not inhibit other MAPKs (Bonny et al., 2001; Borsello et al., 2003; Fig. 1*A*). We hypothesized that in the absence of light, steric hindrance conferred by a constrained Jα helix would prevent JBD from binding and inhibiting JNK, thereby allowing unhindered function of the kinase within the cell. Conversely, on illumination, relaxation of the Jα helix should release this constraint and facilitate kinase inhibition.

### An optically controlled JNK inhibitor assumes an inward-pointing, constrained dark-state and outward-pointing extended lit-state conformation

To validate LOV2-JBD as a light-regulated JNK inhibitor, we used a *dark-state* mutant of LOV2 encoding a C450A mutation in the photo-sensing domain to impose a constrained conformation (Salomon et al., 2000), and a *lit-state* mutant with I539E mutation in the Jα helix, that imposes a relaxed conformation (Harper et al., 2004; Fig. 1*B*). Using homology modeling, we found that both *lit-state* and *dark-state* mutant Cα atoms aligned well to the crystallographic reference structure for LOV2 with RMSDs of 0.52 and 0.57 Å, respectively (Fig. 1*C*). Also, the top five Jα helix models retained six α-helical turns consistent with crystallographic and NMR *A. sativa* LOV2 (Halavaty and Moffat, 2007; Peter et al., 2010). However, the tandem-encoded 11-mer JBD switched from an inward pointing α-helix in the *dark-state* mutant to an extended conformation that projected away from LOV2 in the *lit-state* mutant (Fig. 1*C*). Notably, the relaxed conformation of the *lit-state* mutant JBD was similar to the structure of JIP1(11) bound to JNK1 (Heo et al., 2004), consistent with this being an inhibitory conformation (Fig. 1*D*). Alignment of Cα atoms gave an overall RMSD of 1.68 Å, indicating close alignment between the structures (Fig. 1*C*,*D*). These data predicted that the LOV2-JBD light sensing Jα helix would undergo a conformational change on blue light exposure analogous to that of phototropin-LOV2 (Peter et al., 2010; Fig. 1), leading to a relaxed JBD inhibitor conformation, suitable for trans-inhibition of enzymatic activity (Heo et al., 2004).

### Proof of concept that LOV2-JBD inhibits JNK in response to light

To test experimentally whether LOV2-JBD could be induced by light to inhibit JNK in hippocampal cells, we first measured the phosphorylation of mCherry-NES-Jun as a surrogate reporter of JNK activity in the cytoplasm of transfected cells. Hippocampal neurons displayed elevated phosphorylation of mCherry-NES-Jun, consistent with the high basal activity of JNK in thee cytosol that is characteristic of neurons (Coffey et al., 2000; Coffey, 2014). In cells expressing LOV2-JBD, photoactivation using 458-nm laser substantially reduced JNK activity (Fig. 1*E*). Importantly expression of GFP-LOV2 alone did not alter JNK activity (Fig. 1*F*), indicating that the JBD sequence was required and that LOV2 alone did not affect JNK activity.

### JNK is catalytically active in dendritic spines and can be optically controlled

To visualize real-time JNK activity in neurons, we modified an intramolecular FRET reporter (JNKAR1EV; Komatsu et al., 2011) by substituting mRuby2/Clover for the ECFP/YPET FRET pair to generate a reporter that had increased sensitivity (Extended Data Fig. 2-1*B*), and minimal spectral overlap with LOV2-JBD. Baseline measurements in hippocampal neurons indicated that JNK activity was elevated not only in dendrites, as previously shown (Björkblom et al., 2005), but also in dendritic spines (Fig. 2*A*). After treatment with anisomycin, an activator of JNK (Cano et al., 1994), FRET activity increased further in dendrites and spines (Fig. 2*B-D*), whereas on expression of the Flag-JBD inhibitor, reporter activity decreased by 90% (Fig. 2*E*,*F*). These data indicate that mRuby2-JNKAR1EV-Clover provides a sensitive and specific measure of JNK activity in living neurons. We next used mRuby2-JNKAR1EV-Clover to test optogenetic inhibition of JNK. Photostimulation of LOV2-JBD using 458-nm illumination decreased FRET reporter activity by 50% (Fig. 2*E*,*F*), whereas in cells expressing FRET reporter alone, 458-nm illumination had no effect (control), indicating that light-induced inhibition of JNK FRET reporter activity required LOV2-JBD. In these experiments, we did observe a moderate inhibition of JNK activity in cells expressing LOV2-JBD merely on 488-nm excitation of mRuby2-JNKARIEV-Clover. The extent of inhibition was 30%, as validated also with an independent JNK activity reporter (Extended Data Fig. 2-2).

### JNK activity regulates dendritic spine motility

The effect of JNK on dendritic spine motility was measured using arithmetic difference projection images of GFP-actin generated from a time-series (Fig. 2*G*). Dendritic spines were highly motile in 16-DIV hippocampal neurons, in cells expressing Flag-JBD, spine-head movement was blocked (Fig. 2*G*,*H*). Cells expressing the *lit-state* LOV2-JBD mutant also exhibited reduced motility, whereas neurons expressing the *dark-state* mutant which fails to inhibit JNK (Extended Data Fig. 1-1*A*,*B*), did not show altered dynamics (Fig. 2*G*,*H*). We found that a relatively low illumination of 0.4 mW was sufficient to reduce spine motility (Fig. 2*I*), and we used this “threshold” setting for photoactivation of LOV2-JBD from here on so as to avoid photo-toxic effects.

### JNK acts locally within the spine-head to control spine motility

**Figure 3.**
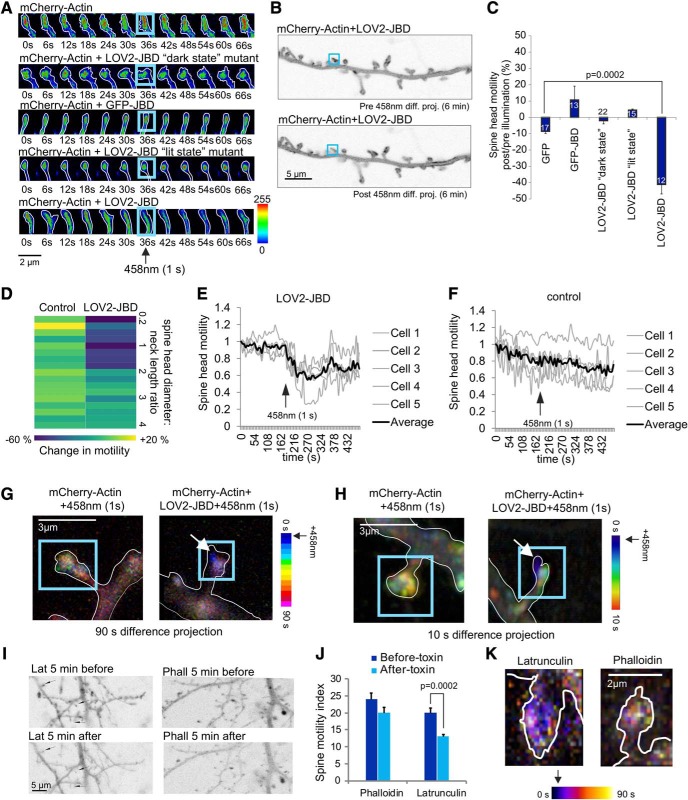
Photoactivation of the LOV2-JBD inhibitor rapidly reduces actin motility in the peripheral domain of the spine. ***A***, Time-lapse sequences from 16-d hippocampal neurons expressing mCherry-actin and LOV2-JBD variants. ROIs (blue squares) encompassing spine-heads were photostimulated for 1 s at 36 s using 0.4 mW of 458-nm light. Spine-head motility was reduced following light only in cells expressing LOV2-JBD. Additional examples are in Extended Data Figure 3-1*A*. ***B***, Arithmetic difference projections of mCherry-actin before (pre) and after (post) 458-nm illumination of the spine (blue box). LOV2-JBD immobilized mCherry-actin in photoactivated spines only. Dendritic shaft mCherry-actin was unchanged. ***C***, Quantitative data on mCherry actin motility. Mean data ± SEM are shown. Spine numbers are indicated on the bars. ***D***, mCherry-actin motility changes are plotted according to spine head diameter:neck length ratio. Data from spines with head diameter:neck length ratios corresponding to “mushroom” spines is in Extended Data Figure 3-1*B*. ***E***, Time-lapse of spine-head mCherry-actin motility shows LOV2-JBD immobilizes actin motility within 6 s of photoactivation. ***F***, Light alone does not alter actin dynamics. For E-F, more than or equal to four spines were measured per cell. ***G***, Color-coded time projections provide spatial information on mCherry-actin motility over 90-s recording. Photoactivation is at 0 s. Images are acquired at 6-s intervals and coded with a unique hue (LUT). Mixed color (white-ish) indicates continued motility over time. Blue indicates no further movement after that time point. LOV2-JBD-expressing neurons display reduced motility following light. ***H***, Higher resolution maximum projections of temporally color-coded dendritic spines are shown from 10-s time lapses acquired at 1-s intervals. These show mCherry-actin is immobilized in the spine periphery. Additional examples with actin footprints and movies generated from the time lapses are in Extended Data Figure 3-1*C*–*E*. ***I***, The effect of latrunculin and phalloidin on dendritic spine motility were tested. Arithmetic projection images before and after 458-nm light indicate effect on actin dynamics. ***J***, Quantitative data from ***I*** indicate that latrunculin reduces actin motility. Measurements are from ≥10 cells and multiple spines from separate experiments. Mean data ± SEM are shown. ***K***, Temporal color coding of spines from neurons treated with latrunculin or phalloidin are shown.

We next exploited the spatiotemporal control of optogenetic inhibition to examine JNK regulation of spine motility in more detail by specifically illuminating the inhibitor in the spine-head. In the absence of 458-nm light, there was rapid displacement of mCherry-actin in the head and neck of the spine and prominent re-shaping or morphing (Fig. 3*A*). Selective ROI photostimulation of LOV2-JBD solely in the spine-head was sufficient to reduce mCherry-actin motility; however, cells expressing the *dark-state* LOV2-JBD mutant remained unaltered (Fig. 3*A-C*). Notably, there was no change in motility observed in the proximal dendrite or in non-illuminated spines (Fig. 3*A-C*), indicating that JNK exerts rapid control of actin movement from within the spine-head (Extended Data Fig. 3-1*A*).

10.1523/ENEURO.0303-19.2019.f3-1 Extended Data Figure 3-1 Photostimulation of LOV2-JBD in dendritic spines rapidly immobilizes spine-head actin in the peripheral domain. ***A***, Time-lapse sequences from 16-d hippocampal neurons expressing mCherry-actin and LOV2-JBD variants as indicated. Cells were stimulated for 1 s with 0.4 mW of 458-nm light. ***B***, The effect on spine motility of LOV2-JBD photoactivation in mushroom spines (head diameter:neck length ratio >3) is shown. Spine motility is measured from arithmetic difference projection ratios (motility after light/motility before light). Optical stimulation of mushroom spines did not alter mCherry-actin motility in spines even when laser power was increased to compensate for larger spine-head volume. At least six spines from four experimental repeats were measured for each condition. Mean data ± SEM are shown. ***C***, High-resolution maximum projections show temporally color-coded dendritic spines from 10-s time lapse from 3D recordings using Airyscan mode, at 1-s intervals. ***D***, Footprints of time lapse sequences are shown for color-coded time projections from cells expressing mCherry-actin with or without LOV2-JBD for spines shown in ***C***. ***E***, Movies generated from 3D Airyscan recordings (1-s interval) of dendritic spines of a cell expressing mCherry-actin with LOV2-JBD as shown in Movie 1: time lapse movie 0-movie 120 s, blue circle depicts ROI of 458-nm illumination. Movie 2, volumetric temporal color coding of a dendritic spine generated from 48 to 58 s with 1-s 458-nm illumination at 48 s. Download Figure 3-1, TIF file.

### JNK controls motility of spines with small “head diameter to neck length” ratios

Mature dendritic spines consist of large, mushroom-shaped protrusions; yet both thin and mushroom spines can appear and disappear throughout adulthood and are important for synaptic plasticity (Holtmaat et al., 2008). To define which spine-type is primarily regulated by JNK, we analyzed spines of different size categories. We defined spines according to the average head diameter to neck length ratio assumed by a given spine during the imaging sequence, as previously (Murase et al., 2016). Interestingly, JNK inhibition reduced motility only in spines that had an average head to neck ratio of between 0.2 and 1.5, corresponding to “thin” and “long thin” spines (Fig. 3*D*). In contrast, photostimulation of LOV2-JBD in mushroom spines (head diameter:neck length ratio >3), did not alter dynamics, even when laser power was increased to account for larger spine-head volume (Extended Data Fig. 3-1*B*). This data indicate that JNK specifically regulates the motility of thin spines.

### JNK exerts rapid control over spine motility

Protein phosphorylation is a highly dynamic process. We therefore measured how quickly a photoactivated JNK inhibitor (LOV2-JBD) could affect actin motility (Fig. 3*E*,*F*). In neurons expressing LOV2-JBD, exposure to a 1-s pulse of 458-nm light was sufficient to rapidly inhibit mCherry-actin motility within 6 s of photostimulation. Motility returned to baseline by 2 min (Fig. 3*E*). We used temporal color coding to obtain spatiotemporal information on actin regulation. Time projection images revealed that optogenetic inhibition of JNK halted actin dynamics by 2 s (blue) in the periphery of the spine and movement did not resume in this region during 90 s of monitoring (Fig. 3*G*,*H*). Interestingly, actin motility decreased in a polarized fashion, starting on one side of the spine-head (blue) and progressing to the opposite side by 5 s (green), whereas the core domain of actin in the spine center remained motile (white) on JNK inhibition (Fig. 3*H*; Extended Data Fig. 3-1*C–E*). These data suggest that JNK controls actin dynamics in the peripheral domain of the spine.

To help understand the contribution of actin dynamics to the spine motility changes measured, we treated neurons with either latrunculin (which binds G-actin and prevents polymerization), or phalloidin (which binds and stabilizes F-actin; Saito, 2009). Latrunculin treatment of neurons reduced spine motility (Fig. 3*I*,*J*) and yielded a characteristic “blue” time projection image suggesting that inhibition of F-actin formation reduced spine dynamics (Fig. 3*K*). In contrast, phalloidin-treated neurons maintained spine motility (Fig. 3*I*,*J*) and showed a distribution of movements throughout the 90-s monitoring (Fig. 3*K*). The temporal coding pattern observed with optogenetic inhibition of JNK is similar to that obtained with latrunculin suggesting dissolution of F-actin.

### JNK is activated by corticosterone leading to internalization of AMPAR and rapid spine retraction

**Figure 4.**
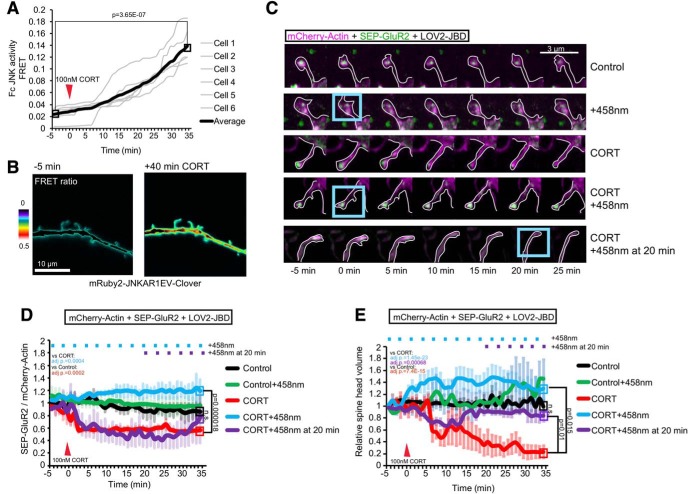
Corticosterone activates JNK and induces SEP-GluR2 removal and spine regression. ***A***, Time-lapse recording of JNK activity in dendritic spines after corticosterone (CORT) application. Normalized Fc FRET is from six cells and more than or equal to four spines per cell. ***B***, Representative FRET ratio images from mRuby2-JNKAR1EV-Clover expressing cells. ***C***, Representative images of time-lapse sequences (***D***, ***E***) from 16-d hippocampal neurons expressing mCherry-actin (magenta), SEP-GluR2 (green), and LOV2-JBD. Cells were treated with CORT (100 nM) at 0 min, and LOV2-JBD was photoactivated using 458-nm 1-s light pulses (using 3% laser power, LSM-880 Airyscan) applied to ROI (blue boxes) at 3-min intervals where indicated (+458 nm), or in lower panels, after a 20 min delay. SEP-GluR2 was imaged using the 488-nm laser (0.8% laser power with LSM-880 Airyscan), to minimize cross-activation of LOV2-JBD. ***D***, Quantitative data on cell surface SEP-GluR2 fluorescence is from eight experiments as depicted in ***C***. ***E***, Estimated spine-head volume is normalized to baseline volume, averaged over 3 min before treatment. Extended Data Figure 4-1 shows experiments repeated as in ***C–E*** with CORT treatment using YFP as an inert filler instead of mCherry-actin. Quantitative data are calculated from eight experiments. Mean data ± SEM are shown. Adjusted *p* values comparing full timelines are from repeated measures one-way ANOVA with Bonferroni correction.

We were interested to know whether JNK regulated spine retraction in the context of synaptic pathology because JNK1 was recently reported to control depressive and anxiety-like behaviors in mice (Hollos et al., 2018; Mohammad et al., 2018). We therefore used corticosterone, the principal glucocorticoid stress hormone in mice, which stimulates retraction of dendritic spines in specific brain regions (Liston and Gan, 2011). JNK activity was measured in hippocampal neurons expressing mRuby2-JNKAR1EV-Clover as earlier (Fig. 2). Corticosterone (100 nM) increased JNK activity within 10 min in dendritic and spine compartments (Fig. 4*A*,*B*).

10.1523/ENEURO.0303-19.2019.f4-1 Extended Data Figure 4-1 Optogenetic inhibition of JNK recovers regressed spines and SEP-GluR2 induced by corticosterone. ***A***, Time-lapse sequences from 16-d hippocampal neurons expressing eYFP-C1 (magenta), SEP-GluR2 (green), and LOV2-JBD. Cells were treated with 100 nM CORT at 0 min; 458-nm light pulses were applied every 3 min where indicated (+458 nm). ***B***, Quantitative data show spine-head volume changes relative to baseline, calculated from six experiments. **C.** Quantitative data show plasma membrane SEP-GluR2 fluorescence normalized to YFP. Data are from six experiments as depicted in ***C***. Adjusted *p* values (written on the graph) are from comparisons of full timelines from multiple experiments using repeated measures one-way ANOVA with Bonferroni correction. Endpoint averages are also shown. Download Figure 4-1, TIF file.

As AMPA receptor removal from the synapse is an early event during spine shrinkage (Hanley, 2014), we next investigated whether corticosterone would affect this process. GluR2 endocytosis was visualized as previously described (Kessels et al., 2009) using a pH-luorin-tagged SEP-GluR2 which loses fluorescence when internalized to the low pH endocytic vesicles. Corticosterone rapidly (within 5 min) reduced levels of SEP-GluR2 fluorescence at the plasma membrane in the spine head. Interestingly, this was completely blocked when JNK was inhibited by photoactivation (Fig. 4*C*,*D*). Moreover, photoactivation of LOV2-JBD prevented SEP-GluR2 internalization even when started 20 min after addition of corticosterone (Fig. 4*C*,*D*).

We also measured the effect of corticosterone on spine-head volume. This decreased within 10 min of treatment with the glucocorticoid (Fig. 4*C*,*E*). Optogenetic inhibition of JNK rescued spine retraction even when light was applied 20 min after corticosterone (Fig. 4*C*,*E*). These data suggest that JNK controls GluR2 removal and regression of spines in response to corticosterone. To assess if effects of actin overexpression would skew the estimate of motility measurements, we also measured volumes and GluR2 endocytosis levels using soluble YFP. Corticosterone induced rapid reduction of SEP-GluR2 fluorescence and spine-head volume, both of which were rescued on optogenetic inhibition of JNK (Extended Data Fig. 4-1).

### NMDA activates JNK and induces rapid spine shrinkage

**Figure 5.**
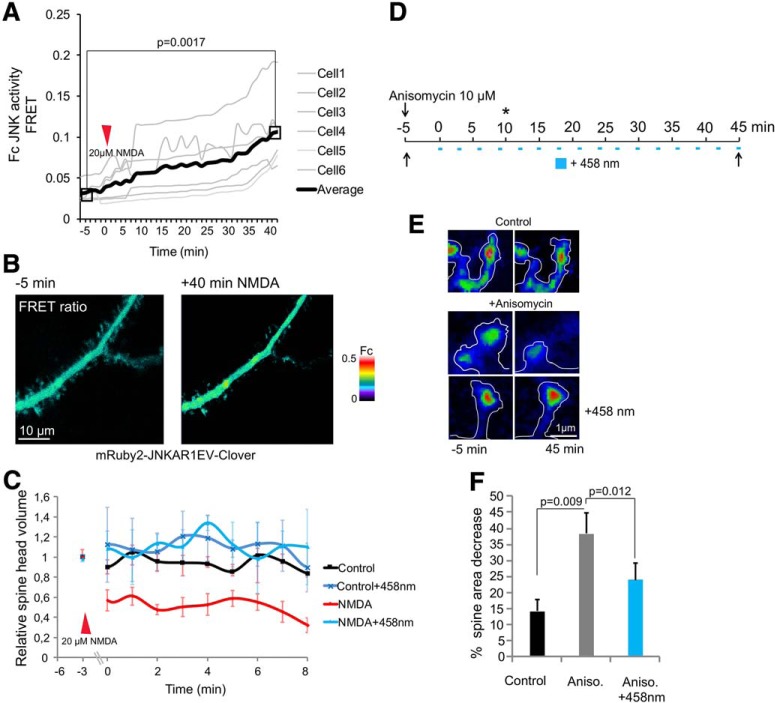
NMDA activates spine-head JNK and induces retraction. ***A***, Time lapse of JNK activity from mRuby2-JNKAR-1EV-Clover FRET reporter. ROI FRET (Fc) was measured from spines of 16-d hippocampal neurons before and after NMDA (20 μM). ***B***, Representative FRET images before and after NMDA. ***C***, The estimated spine-head volume from neurons expressing mCherry-actin and LOV2-JBD before (–3 min) or after treatment with NMDA are shown. Continuous photoactivation of LOV2-JBD was achieved using 1-s pulses of 458-nm laser (3% power) at 3-min intervals. LOV2-JBD photoactivation prevented NMDA-induced spine retraction. ***D***, The experimental setup for anisomycin (10 μM) treatment and photoactivation timeline is shown. The “*” indicates time point at which JNK was activated. ***E***, Neurons were treated with or without anisomycin and spine volume measured at 45 min (as described in D). Anisomycin induced spine retraction which was prevented by LOV2-JBD photoactivation. ***F***, Mean data ± SEM from 20–24 spines/treatment is shown. Adjusted *p* values comparing full timelines are shown from repeated measures one-way ANOVA with Bonferroni correction.

The glutamatergic system is strongly implicated in depression (Sanacora et al., 2012). We therefore examined JNK regulation by NMDA using FRET as earlier (Figs. 2*B*, 4*A*). NMDA activated JNK within minutes in dendritic and spine compartments (Fig. 5*A*,*B*). Three-minute bath application of NMDA followed by 10-min rest induced rapid spine shrinkage that was prevented by photostimulation of LOV2-JBD (Fig. 5*C*). We next tested anisomycin, a ribotoxin that strongly activates JNK (Fig. 2*B*). Anisomycin treatment induced >40% reduction of spine area (Fig. 5*D-F*).

### Ketamine inhibits JNK and prevents spine retraction and SEP-GluR2 receptor removal

**Figure 6.**
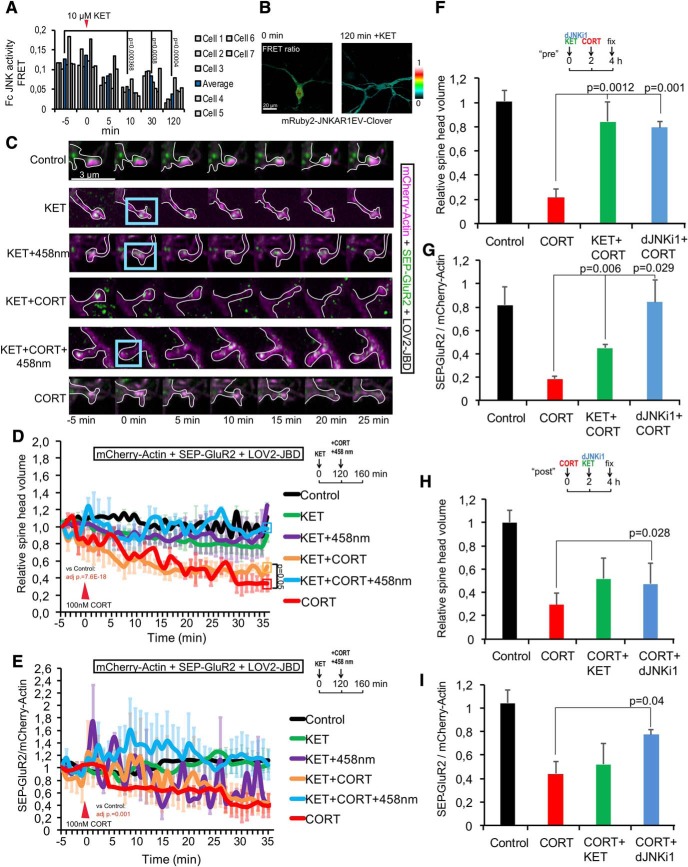
Ketamine inhibits JNK and prevents spine regression when given prophylactically, whereas JNK inhibition provides more robust rescue even 2 h after CORT. ***A***, We tested the effect of ketamine (10 μM) on JNK activity in 16-d hippocampal neurons using the mRuby2-JNKAR1EV-Clover FRET reporter. FC FRET responses in spine-head ROIs are from 7 separate experiments plotted individually; *p* values are shown from repeated measures one-way ANOVA with Bonferroni correction. Ketamine inhibited JNK activity, visible by 10 min postaddition. ***B***, The effect was long lasting. Representative FRET ratio images show mRuby2-JNKAR1EV-Clover JNK activity reporter FRET before and 2 h following ketamine. ***C***, Representative images of 16-d hippocampal neurons expressing mCherry-actin (magenta), SEP-GluR2 (green) ± LOV2-JBD. Cells were stimulated with 100 nM corticosterone (CORT) or 10 μM ketamine (KET), as indicated. Blue boxes indicate ROI where 458-nm light was applied (1-s pulses at 3-min intervals) to photoactivate LOV2-JBD. ***D***, Estimated spine volume changes were calculated from multiple images as shown in ***C***, normalized to baseline. Eight cells, four spines per cell were used. ***E***, Quantitative data of cell surface SEP-GluR2 (SEP-Glur2 fluorescence/mCherry-actin fluorescence) are shown from the same cells as in ***D***. Measurements are from eight experiments. ***F***, Dendritic spine volume changes were calculated from cells pretreated with 10 μM ketamine or 10 μM DJNKI-1 (to inhibit JNK) 2 h before addition of CORT (100 nM) for 2 h. ***G***, Cell surface SEP-GluR2 levels (SEP-GluR2 fluorescence/mCherry-actin fluorescence) was measured from the same cells. ***H***, Estimated spine volume changes were from more than or equal to eight experiments; 16-d neurons were treated with CORT (100 nM) for 2 h followed by KET (10 μM) or DJNKI-1 (10 μM). Estimated spine volume was measured and volume at 4 h was expressed relative to control. ***I***, Cell surface GluR2 levels (SEP-GluR2/mCherry-actin) were calculated from the same cells. Mean data ± SEM are shown. Adjusted *p* values are shown from repeated measures one-way ANOVA with Bonferroni correction.

As NMDA is strongly activated by JNK (Fig. 5), we tested whether the NMDA receptor antagonist, fast acting anti-depressant ketamine, altered JNK activity. Indeed, JNK activity was significantly reduced after 2 h of ketamine treatment (Fig. 6*A*,*B*). However, ketamine did not prevent SEP-GluR2 removal from spines, nor did it prevent spine retraction in response to corticosterone at this time point (Fig. 6*C-E*). Because the timeline of these ketamine treatments was only 40 min, less than the 2 h required to inhibit JNK (Fig. 6*A*,*B*), we tested whether a 2-h pre-treatment with ketamine would prevent corticosterone-induced spine retraction. This prevented corticosterone-induced spine shrinkage and inhibited GluR2 removal (Fig. 6*F*,*G*). Finally, we examined whether treatment with ketamine or DJNKI-1 2 h following corticosterone could rescue GluR2 internalization and spine retraction. Interestingly, inhibition of JNK 2 h after corticosterone recovered spine volume and GluR2 homeostasis (Fig. 6*H*,*I*). Ketamine treatment did not rescue at this timepoint, likely because there was insufficient time to achieve robust JNK inhibition. These data show that prophylactic treatment with ketamine or JNK inhibitor prevents spine retraction and GluR2 removal in response to corticosterone. Inhibition of JNK supports spine regrowth and rescues corticosterone effects on molecular events in spines even in the continued presence of corticosterone.

## Discussion

Structural plasticity of dendritic spines facilitates elimination and regrowth of synapses and provides a fundamental process for adaptive neurotransmission. Understanding the underlying mechanism is relevant for memory and learning but also for pathologies such as depression, where significant spine loss is a hallmark (Duman and Duman, 2015; Musazzi et al., 2015; Varidaki et al., 2016). Here, we identify using an optogenetic approach, that catalytically active JNK controls spine stability from within the spine-head. Using arithmetic difference projections to quantify mCherry-actin displacements over time, we observe a clear reduction of movement in spines commencing within seconds of LOV2-JBD photostimulation to inhibit JNK solely in the spine-head. This rapid response indicates a proximal mechanism whereby JNK acts directly in the spine to suppress motility. Full recovery of spine movement after photostimulation is ended takes ∼2 min, consistent with the photo-cycle of LOV2-JBD which lasts ∼80 s, after which a constrained state for LOV2-JBD is expected to reestablish (Zayner and Sosnick, 2014).

We show that JNK predominantly regulates spines with smaller head to neck length ratios, suggesting a more pronounced control of thin spines. This spine class emerge as motile protrusions during new synapse formation, enriched with dynamic actin (Engert and Bonhoeffer, 1999; Lendvai et al., 2000; Matsuzaki et al., 2004; Cingolani and Goda, 2008), and is prominent throughout life during synapse formation (Fischer et al., 2000; Holtmaat et al., 2008). Our data establish that physiologically active JNK controls actin dynamics locally in thin spines.

We show using temporal color coding that optogenetic inhibition of JNK alters the actin pool at the periphery of the spine rather than in the central region closer to the base of the spine and neck. The peripheral pool of F-actin in this region has been shown to turnover rapidly with a half-life of around 10 s compared to the stable pool of cross linked F-actin at the spine center which has a half-life of 17 min (Honkura et al., 2008; Kasai et al., 2010; Bertling et al., 2016). Interestingly, the dynamic F-actin pool in the peripheral domain that JNK controls is the same pool that regulates AMPA receptor membrane trafficking (Cingolani et al., 2008; Honkura et al., 2008), a central event during synaptic plasticity changes (Huganir and Nicoll, 2013). Consistent with this, we find that corticosterone-activated JNK facilitates rapid (within 5 min) removal of SEP-GluR2 from the spine-head membrane before spine retraction. This is blocked on optogenetic inhibition of JNK in spines. As the GluR2 subunit is associated predominantly with functional synapses (Passafaro et al., 2001), these thin spines that are controlled by JNK most likely represent immature synapses. Thus, our data uncovers that JNK activity reduces spine-head actin dynamics in the peripheral domain which is permissive for receptor removal. This mechanism may be relevant for LTD and impaired short-term memory regulation by JNK where AMPA receptor removal plays a central role in downregulation of synaptic function (Bevilaqua et al., 2003; Li et al., 2007; Myers et al., 2012).

Prolonged hyperactivity of the Hypothalamic-Pituitary-Adrenal (HPA) axis leading to elevated glucocorticoids is associated with psychiatric disorders, in particular depression and anxiety (Malhi and Mann, 2018). Both endocrine and glutamate stress have been shown to induce synapse regression and dendritic atrophy that can lead to maladaptive circuit changes and depressive behaviors (Musazzi et al., 2011; Bennett and Thomas, 2014; Duman and Duman, 2015). In mice, chronic corticosterone is used to model depression, although even acute stress can induce long-term pathologic changes at synapses (Musazzi et al., 2018). Corticosterone treatment of mice was previously shown to activate JNK in the hippocampus (Solas et al., 2013). Here, using real-time FRET to measure spatiotemporal JNK activation by corticosterone, we find that spine-head JNK is activated within 10 min of treatment, followed by a second phase of activation after 20 min. This could be a consequence of increased glutamate efflux which occurs following corticosterone (Musazzi et al., 2010, 2017; Sanacora et al., 2012; Treccani et al., 2014), as we show that the NMDA receptor activates spine-head JNK within this timeframe. The downstream effect of corticosterone-activated JNK is the rapid removal of SEP-GluR2 from synapses which occurs by 5 min, followed by spine retraction commencing 10 min after corticosterone. Moreover, spine regrowth after corticosterone treatment can be recovered in spines where JNK is inhibited, up to 20 min after corticosterone addition. These data indicate that JNK activity regulates early events leading to spine retraction following corticosterone stress, whereas inhibition of JNK promotes spine regrowth, even under conditions of maintained endocrine stress.

Strengthening and weakening of synaptic transmission is controlled by NMDA. High-frequency stimulation of the NMDAR coincident with pre-synaptic activity induces spine growth and synapse strengthening, whereas NMDA in the context of chemical-LTD induces spine shrinkage (Zhou et al., 2004). Here, we use the chemical LTD protocol (Lee et al., 1998), which is believed to mimic low level long lasting NMDA receptor stimulation leading to down regulation of postsynaptic AMPARs and synapse regression (Lee et al., 1998; Lüscher and Malenka, 2012). This LTD protocol induces a reduction in relative spine-head volume within 3 min, which is completely rescued on LOV2-JBD photoactivation. These results suggest that JNK activation locally within the spine may facilitate LTD. Consistent with this possibility, JNK1 has been previously implicated in LTD both in inhibitor DJNKI1 and *Jnk1-/-* knockout studies (Ge et al., 2007; Li et al., 2007), although the nuclear substrate c-Jun is not involved as Jun-AA mice undergo normal LTD (Seo et al., 2012).

We find that either ketamine or JNK inhibition promote hippocampal neuron spine recovery and SEP-GluR2 receptor trafficking in response to corticosterone. Moreover, we identify that ketamine treatment leads to run-down of JNK activity in a relatively slow and phasic manner. Nonetheless, JNK inhibition may be a critical downstream mediator of ketamine action. The kinetics of ketamine action on spines lags behind that of JNK inhibition, consistent with JNK inhibition being a necessary downstream event. Clearly more work will be needed to establish whether JNK inhibition contributes to ketamine action on spines in hippocampus and to determine whether this action serves any consequence in circuit remodeling. Although ketamine regulation of spines in prefrontal cortex has been dissociated from its behavioral effect in mice (Moda-Sava et al., 2019), the relevance of ketamine or JNK inhibition on spine dynamics in hippocampus has not been studied in the context of depression. It is worth noting however, that like ketamine, *Jnk1* deletion or JNK inhibition using the same peptide inhibitor that is encoded in our optogenetic tool, lowers anxiety- and depressive-like behaviors in mice (Hollos et al., 2018; Mohammad et al., 2018).

Optogenetic inhibition of JNK has allowed us to identify that JNK controls the dynamics of spine-head actin from within the spine where it controls corticosterone-induced and chemical-LTD-induced spine plasticity. These findings describe a previously unknown role for JNK in structural plasticity of synapses in the context of endocrine stress where JNK triggers rapid receptor removal and spine regression.

## Synthesis

Reviewing Editor: Orly Reiner, Weizmann Institute of Science Decisions are customarily a result of the Reviewing Editor and the peer reviewers coming together and discussing their recommendations until a consensus is reached. When revisions are invited, a fact-based synthesis statement explaining their decision and outlining what is needed to prepare a revision will be listed below. The following reviewer(s) agreed to reveal their identity: Menahem Segal, Simon Wiegert.

The authors of the present manuscript investigate the role of C-Jun-terminal kinase (JNK) in structural and functional spine plasticity. They developed an optically controlled inhibitor of JNK based on the photosensitive LOV2 protein and a JNK binding domain (JBD) and they further improved a JNK FRET sensor to investigate JNK signaling with live cell imaging. Using these and other tools, the authors show that JNK activity triggers AMPA receptor internalization and subsequent spine shrinkage. Moreover, they demonstrate that these processes, which can be triggered by NMDA-mediated excitotoxicity or corticosterone, can be prevented by optogenetic or pharmacological inhibition of JNK. Thus, the present study establishes a central role of JNK as regulator of spine stability.

The authors combine molecular and imaging tools to zoom in on dendritic spines and detect changes in several molecular entities following exposure to drugs and to photoactivation of JNK blockade. The results are interesting and novel.

The authors are requested to address the following issues:

Reviewer 1:

First, the authors focus on dendritic spines, but I assume they shine light also on the entire cell/dendrites. Do they find any morphological/structural changes in dendrites? If so, is it possible that the changes they observe in spines are secondary to dendritic changes? In any case, they should address such changes too (there should be changes in dendrites, seen by others).

Second, for some reason, they focus in both the introduction and discussion on a possible relevance of these results to stress and depression. There is a long way between cellular stress mechanisms in cultured hippocampal neurons and behavioral stress and depression in animals and humans, which may take place in structures other than the hippocampus. Depression is a far more complex phenomenon than a simple consequence of spine shrinkage, which underlies many other neuronal functions.

By the same token, the use of NMDA is a bit complicated. Others have shown that activation of the NMDA receptor may cause spine formation, inflation or conversion of thin to mushroom spines. The difference between the present and previous results should be discussed.

Interestingly, they report that JNK modifies only thin spines. This may underlie some differences between the present and previous results on the involvement of JNK in plasticity. Which reminds me that they fail to cite and discuss some earlier reports on JNK and dendritic spines and plasticity (e.g. Seo J. et al 2012, Farias et al. 2009).

In some of the figures (e.g. Fig 4C), the spines are long (>3um) and thin. Do you distinguish between thin spines and filopodia? Others suggest that filopodia are far more motile than spines. Do you see a distinction between these categories? Obviously, if spine head volume (Fig 4E) is very small, a change may be large and possibly significant, just because the initial value is low.

It is not clear how you define the boundary of the spine head (e.g. Fig 5E). The boundary line does not overlap with the colored image of the spine.

Reviewer 2:

First, the FRET sensor requires excitation at 488 nm, which also activates LOV2. Thus, all FRET imaging experiments using LOV2-JBD potentially activate the inhibitor already under baseline conditions. The authors show in Fig. 2I that they get full activation of LOV2-JBD at 450 uJ/mm^2 of 458 n light, which corresponds to 3% of maximal power of the 458 nm laser. Unfortunately, they do not specify the irradiance used for FRET imaging. Instead, only percentages are presented for the 488 and 543 nm lasers. This information is useless and irradiance has to be indicated. Assuming that the 488 nm laser has similar power as the 458 nm laser, activation of LOV2 at 6% 488 nm is very likely. This may explain the lower FRET values before 458 nm illumination compared to control as seen in Figs. 1E and 2F. There needs to be a thorough characterization of LOV2-JBD activation by 488 nm illumination. In addition, the authors need to better quantify dark activity of the tool. It is not clear to what extent the overexpression of LOV2-LBD without any illumination affects JNK signaling.

Next, the authors report spine head volume analyses in multiple figures. However, I do not understand how these measurements were done. None of the reported fluorophores in this study is suitable for spine volume measurements, as they do not uniformly distribute throughout the cytoplasm (e.g. mCherry-actin, SEP-GluA2) or their intensity is not stable over time (e.g. FRET sensor, SEP-GluA2). For example, in Fig. 4E spine volume was measured in cells expressing mCherry-actin, SEP-GluA2 and LOV2-JBD, none of which is suitable to measure spine volumes. The authors need to provide measurements with an inert volume-filling fluorophore. This also accounts for the normalization of SEP-GluA2 measurements to mCherry-actin. Actin itself will be affected by some of the treatments, thus compromising the estimate of AMPAR surface trafficking. Also here, a normalization against a simple fluorophore is required. Related to this, in figure 3, the authors show reduction of spine head motility using mCherry-actin. Again, if the distribution of actin itself is affected, this will skew the estimate of spine head motility. Moreover, from fig. 3A it looks like the spine was illuminated only in the last condition (LOV2-JBD) but not in the four other control experiments. Thus, it is not clear whether illumination itself had an effect on mCherry-actin (e.g. bleaching).

Other concerns are listed below.

In figure 4A the authors show changes in JNK-FRET in a time series experiments where the cells are stimulated with corticosterone. However, the FRET signal is increasing already during baseline and the slope seems to change only 10-15 min after corticosterone application. In Fig. 2B such an increase in baseline signal is not visible. How is this explained? As it is presented, the experiment does not unequivocally reveal the corticosterone effects on JNK activity.

As opposed to what is stated in the main text, the data shown in fig. 4D do NOT indicate that optogenetic activation of LOV2-JBD rescues corticosterone-mediated SEP-GluA2 internalization. First, there seems to be no effect of corticosterone in the first place (compare to red curve). Second, the SEP-GluA2 levels are down to the same value as the corticosterone condition alone (red curve) after 40 min. I am also not convinced that optogenetic stimulation after 20 min led to a rescue. The error bars of the purple curve are very large, making it very unlikely that it differs from the red curve (also see my remarks on statistics below).

In figure 6 the baseline is completely missing. Moreover, this entire figure is very difficult to interpret. Do the authors try to convey the message that 2h pretreatment with ketamine reverts corticosterone-mediated spine shrinkage 2 hours after corticosterone application, but not 40 min after? Why did the authors use optogenetic stimulation in fig. 6 D,E but pharmacology in fig. 6F-H to inhibit JNK? dJNKi1 was used nowhere else in this study. Why is it used here? Further, I assume that the axis labels “KET” and “dJNKi1” in fig. 6F-H in fact mean “KET+CORT” and “dJNKi1+CORT” (and the other way round), like in fig. 6 D,E? With the missing baseline, the inappropriate spine head volume measurements and SEP-GluA2-normalization, I have doubts that the long-term effects of ketamine can be properly interpreted.

Finally, essential statistical analyses are missing or not applied properly. Statistical analysis is missing for figures 4 D,E; 5 C; 6 D,E. Moreover, a t-test cannot be used if more than two groups are present, as in fig. 5 F. Since this figure represents parts of a population, a chi square test would be more appropriate.

## Author Response

Synthesis Statement for Author (Required)

The authors of the present manuscript investigate the role of C-Jun-terminal kinase (JNK) in structural and functional spine plasticity. They developed an optically controlled inhibitor of JNK based on the photosensitive LOV2 protein and a JNK binding domain (JBD) and they further improved a JNK FRET sensor to investigate JNK signalling with live cell imaging. Using these and other tools, the authors show that JNK activity triggers AMPA receptor internalization and subsequent spine shrinkage. Moreover, they demonstrate that these processes, which can be triggered by NMDA-mediated excitotoxicity or corticosterone, can be prevented by optogenetic or pharmacological inhibition of JNK. Thus, the present study establishes a central role of JNK as regulator of spine stability.

The authors combine molecular and imaging tools to zoom in on dendritic spines and detect changes in several molecular entities following exposure to drugs and to photoactivation of JNK blockade. The results are interesting and novel.

The authors are requested to address the following issues:

Reviewer 1:

First, the authors focus on dendritic spines, but I assume they shine light also on the entire cell/dendrites. Do they find any morphological/structural changes in dendrites? If so, is it possible that the changes they observe in spines are secondary to dendritic changes? In any case, they should address such changes too (there should be changes in dendrites, seen by others).

When we examine the effect of JNK inhibition on spines, we do not shine light on the entire dendrite. We illuminate the spine head only. This is done using confocal r.o.i. laser illumination (Fig. 3-6). We developed this optogenetic inhibitor precisely to enable spatial control of JNK within the spine head. The illuminated region is indicated by a blue box. We have made these boxes more prominent in the images to help avoid misunderstanding. We think that it is very unlikely that the changes observed in spines are secondary to a change in dendrites. For example, in Fig.3 A, B, C selective photoactivation of LOV2-JBD in spines leads to actin dynamics changes only in spines, not in dendrites. Also, the timeframe of JNK-induced actin changes in the spine head is very fast (<40s, Fig. 3E). So it seems unlikely that JNK is inhibited in spines and leaves the spine to induce a change in dendrites that in turn feeds back to alter spine actin. Thank you for pointing out literature on JNK and dendrites. Key references are now cited in the introduction regarding JNK regulation of dendrites.

Second, for some reason, they focus in both the introduction and discussion on a possible relevance of these results to stress and depression. There is a long way between cellular stress mechanisms in cultured hippocampal neurons and behavioral stress and depression in animals and humans, which may take place in structures other than the hippocampus. Depression is a far more complex phenomenon than a simple consequence of spine shrinkage, which underlies many other neuronal functions.

We appreciate this comment and have toned down the association with “depression” in the introduction and discussion. We have tried to convey better the relevance of spine shrinkage, a fundamental aspect of synaptic plasticity, for all kinds of neurotransmission and related functions by adding more references on synaptic plasticity and memory for instance. Nonetheless, this study does focus on stressors that are classically used to model depressive disorders rather than LTP/LTD specifically i.e. the fast-acting anti-depressant ketamine and corticosterone. Therefore we do think that some discussion about spine loss and depressive disorders is reasonable, also taking into consideration the overt behavioural phenotype exhibited by Jnk1-/- mice in this context (Mohammad et al., 2018). Last but not least, we identify here for the first time that inhibition of JNK is downstream of ketamine and may be relevant for its anti-depressant action. But the point is absolutely taken and we hope the text is more balanced now.

By the same token the use of NMDA is a bit complicated. Others have shown that activation of the NMDA receptor may cause spine formation, inflation or conversion of thin to mushroom spines. The difference between the present and previous results should be discussed.

We agree that NMDA is complicated. On the one hand NMDA can induce spine growth and synapse strengthening, whereas NMDA in the context of chemical-long-term depression induces spine shrinkage (Zhou et al., 2004). Here we are using the chemical long-term depression model (Lee et al., 1998) which is believed to mimic the low level longer lasting NMDA receptor stimulation leading to down regulation of post-synaptic AMPARs (Lüscher and Malenka, 2012). There is a variance in literature reporting how many applications of NMDA reliably reproduce LTD and synapse loss (Henson et al., 2017) however in our hands this works as originally described in Lee et al. We have added a paragraph to the discussion on LTD and JNK and explaining the LTD/LTP dichotomy.

Interestingly, they report that JNK modifies only thin spines. This may underlie some differences between the present and previous results on the involvement of JNK in plasticity. Which reminds me that they fail to cite and discuss some earlier reports on JNK and dendritic spines and plasticity (e.g. Seo J. et al 2012, Farias et al. 2009).

Thank you for suggesting these references. We appreciate the opportunity to add other key studies that establish causality between JNK and spine regulation (knockout/inhibitor approaches). These references are now included (Curran et al., 2003; Costello and Herron, 2004; Sherrin et al., 2010; Sherrin et al., 2011). For the Farias study we did not find evidence that showed a causal link between JNK and Wnt5 regulation of spines. We also added Yang et al. 2007 and Xei et al., 2007 to the discussion of LTD.

In some of the figures (e.g. Fig 4C), the spines are long (>3um) and thin. Do you distinguish between thin spines and filopodia? Others suggest that filopodia are far more motile than spines. Do you see a distinction between these categories?

In this study we did not include filopodia in the analysis, i.e. very long thin protrusions (>3μm) that lack a visible spine head. Only true spines, with a head broader than the neck were analysed. The effect of JNK inhibition is also present in mushroom spines, but to a lesser extent (Fig. 3D). We see a distinction, largest effect with spine head diameter:neck length ratio 0.2-2, which accounts for 'thin' and 'long-thin' categories, but not filopodia.

Obviously, if spine head volume (Fig. 4E) is very small, a change may be large and possibly significant, just because the initial value is low.

We have done a correlation analysis to determine if spine head volume correlates with the extent of spine shrinkage and while there is a trend towards a negative correlation in CORT treated neurons (R=-0.16) however this correlation was not significant and a power analysis indicated that n=100 would be needed to make it significant. We would like to point out that the “estimated spine volume changes” shown in figure 4 and 6 are extent change in volume relative to a baseline average volume measured over 3 min before treatment.

It is not clear how you define the boundary of the spine head (e.g. Fig 5E). The boundary line does not overlap with the colored image of the spine.

Boundary lines were drawn manually around the maximum projection image of actin over the entire imaging time. Only signal with at least 10 intensity values higher than background was considered positive for actin. This information is now added to the methods section, page 11, lines 12-14. In Fig 5E, we noticed the boundaries shifted perhaps during editing. We have now fixed this.

Reviewer 2:

First, the FRET sensor requires excitation at 488 nm, which also activates LOV2. Thus, all FRET imaging experiments using LOV2-JBD potentially activate the inhibitor already under baseline conditions. The authors show in Fig. 2I that they get full activation of LOV2-JBD at 450 uJ/mm^2 of 458 n light, which corresponds to 3% of maximal power of the 458 nm laser. Unfortunately, they do not specify the irradiance used for FRET imaging. Instead, only percentages are presented for the 488 and 543 nm lasers. This information is useless and irradiance has to be indicated. Assuming that the 488 nm laser has similar power as the 458 nm laser, activation of LOV2 at 6% 488 nm is very likely. This may explain the lower FRET values before 458 nm illumination compared to control as seen in Figs. 1E and 2F. There needs to be a thorough characterization of LOV2-JBD activation by 488 nm illumination.

Firstly, we would like to state that although the FRET probe is used throughout this study, Fig. 2E, F is the only place where it is combined with LOV2-JBD. So the reviewer statement that “all experiments using LOV2-JBD potentially activate the inhibitor under baseline conditions” is referring only to one experiment. Secondly, 3% and not 6% laser power was used for the FRET data. We omitted to add this laser power in the methods, it is added now.

Regarding cross talk during FRET illumination, 488 nm illumination does cause ~30% inhibition of baseline JNKAR FRET in this experiment (Fig. 2F) as was discussed in the original manuscript (Page 13 line 4-9). However, the absorption spectra of LOV2 at 488 nm is 50-60% lower than when illuminated with 458 nm (e.g Solomon et al., 2000; Gauden et al., 2004). Also, studies using LOV2 optogenetic tools in mammalian cells report a weaker effect using 488 nm laser line compared to 458 nm (Wu et al., 2011).

We did additional experiments to measure the irradiance levels at 488nm as the reviewer suggested. When using either 458nm or 488nm lines of the 4-line argon laser on the Zeiss-780 at 3% power, the irradiance measured at the 63x objective was 0.4 mW. Thus for FRET probe illumination, the same irradiance was used (0.4 mW) as for 458nm LOV2 illumination. Theoretically, we would expect a 60% reduced LOV2-JBD activation at 488nm. We tested this directly using an independent readout of JNK activity (P-NES-Jun staining intensity) in cells expressing LOV2-JBD+mcherry-NES-Jun, illuminating with 0.4mW of 488nm laser line. This data shows a 30% inhibition of JNK activity (phospho-NES-Jun phosphorylation) compared to 60% inhibition when using the same irradiance and 458 nm (new data is in Extended Fig. 2-2.), in line with what we show in Fig. 2F and also in line with the predicted value based on decreased absorption spectra at 488. Furthermore, upon 458 nm activation of LOV2-JBD, there is a further 50% inhibition of FRET activity. Thus, although there is a moderate baseline inhibition of LOV2-JBD upon FRET excitation, this does not preclude its use as there is still a substantial further effect with 458 illumination. The FRET probe is useful because of the additional spatial information it brings. We apologise for the lack of clarity in the methods.

In addition, the authors need to better quantify dark activity of the tool. It is not clear to what extent the overexpression of LOV2-LBD without any illumination affects JNK signalling.

Dark activity, including non-illuminated LOV2-JBD, LOV2WT and dark state mutant LOV2-JBD was characterized in Figure 1E and F and in Extended Figure 1-1A and B. For example in Fig.1E, JNK activity (P-Jun intensity in cells expressing mCherry-NES-Jun reporter) is compared in cells with and without LOV2-JBD. There is no significant difference, however a trend towards decreased activity. We have relabelled this figure to improve clarity as perhaps this point was missed.

Next, the authors report spine head volume analyses in multiple figures. However, I do not understand how these measurements were done. None of the reported fluorophores in this study is suitable for spine volume measurements, as they do not uniformly distribute throughout the cytoplasm (e.g. mCherry-actin, SEP-GluA2) or their intensity is not stable over time (e.g. FRET sensor, SEP-GluA2). For example, in Fig. 4E spine volume was measured in cells expressing mCherry-actin, SEP-GluA2 and LOV2-JBD, none of which is suitable to measure spine volumes. The authors need to provide measurements with an inert volume-filling fluorophore. This also accounts for the normalization of SEP-GluA2 measurements to mCherry-actin. Actin itself will be affected by some of the treatments, thus compromising the estimate of AMPAR surface trafficking. Also here, a normalization against a simple fluorophore is required. Related to this, in figure 3, the authors show reduction of spine head motility using mCherry-actin. Again, if the distribution of actin itself is affected, this will skew the estimate of spine head motility.

We have expanded the methods description for this part as follows:

“To analyse spine head volume, r.o.i. voxels were drawn encompassing the entire volume occupied by visible mCherry-Actin or YFP in the spine head throughout the time lapse as previously (Basu, 2018; Hruska, 2018), ensuring that the entire 4D footprint was included in the analysis. The z-depth was 4 μm and x, y dimensions varied according to individual spine size. Spines were imaged in 3D with a z-section of 0.2 μm scan speed of 3 frames per s. Relative changes in dendritic spine volume were estimated from maximum intensity projections from these images. To calculate a baseline spine volume, maximum projections from 3 min of image acquisition prior to treatment were averaged. Subsequent recordings were normalized to this value. ”

Regarding the comment on the use of mCherry-actin to estimate spine volume, we would like to point out that this is a very commonly used approach, if not the most common. We like it because it provides the best signal to noise ratio in labelled spines and allows imaging with low level light thereby minimizing phototoxicity (Hluschenko, 2016; Melak et al., 2017). Spine head volume is proportional to the overall amount of F-actin in spines (correlation coefficients of 0.95 and 0.97; Honkura et al., Cell 2008), therefore although it can be argues that it is not a homogenous space filling marker, it is a surrogate marker. Space filler markers such as GFP or YFP which do not concentrate in spines give a relatively dim signal (the cytoplasmic volume is after all small). Thus to achieve comparable signal:noise, requires higher level overexpression. No single method is perfect. To address this question, and validate the responses obtained with actin, we did new experiments in 16 DIV hippocampal neurons expressing YFP as a filler. This new data (Extended Fig. 4-1) confirms our original finding that CORT induces spine shrinkage and LOV2-JBD rescues this. The data with YFP is more noisy than that obtained with mCherry-Actin likely due to lower signal:noise associated with dimmer images.

Moreover, from fig. 3A it looks like the spine was illuminated only in the last condition (LOV2-JBD) but not in the four other control experiments. Thus, it is not clear whether illumination itself had an effect on mCherry-actin (e.g. bleaching).

We have now moved the representative images that the reviewer is asking for from the extended data to the main figure. However, the quantitative data for these is in Figure 3 C, F, G (left panel), H (left panel) showing quantitative changes before and after light.

Other concerns are listed below.

In figure 4A, the authors show changes in JNK-FRET in time series experiments where the cells are stimulated with corticosterone. However, the FRET signal is increasing already during baseline and the slope seems to change only 10-15 min after corticosterone application. In Fig. 2B such an increase in baseline signal is not visible. How is this explained? As it is presented, the experiment does not unequivocally reveal the corticosterone effects on JNK activity.

The increase seen in the averaged 5 min baseline is very minor and it is not significant (t-5 vs t0 data points t-test p=0.52). The variance is likely due to cell to cell heterogeneity.

As opposed to what is stated in the main text, the data shown in Fig. 4D do NOT indicate that optogenetic activation of LOV2-JBD rescues corticosterone-mediated SEP-GluA2 internalization. First, there seems to be no effect of corticosterone in the first place (compare to red curve). Second, the SEP-GluA2 levels are down to the same value as the corticosterone condition alone (red curve) after 40 min.

The reviewer is possibly looking at the wrong figure. There is a clear effect of CORT on SepGluR2 internalisation (Fig. 4D, red). This large effect is visible by eye and there is a complete rescue upon 458nm illumination of LOV2-JBD (blue). The statistical analysis is done using one-way ANOVA followed by Bonferroni post-hoc test for multiple comparisons. Secondly, the control levels to not decrease to CORT levels (red line).

I am also not convinced that optogenetic stimulation after 20 min led to a rescue. The error bars of the purple curve are very large, making it very unlikely that it differs from the red curve (also see my remarks on statistics below).

It is not clear which figure the reviewer refers to here. What we have claimed is that optogenetic stimulation rescues spine volume change 20 min after corticosterone (Fig. 4C, E). Significance is determined by one-way ANOVA whole timeline analysis with post-hoc testing and endpoint analysis with ANOVA.

In Fig. 6, the baseline is completely missing. Moreover, this entire figure is very difficult to interpret. Do the authors try to convey the message that 2h pre-treatment with ketamine reverts corticosterone-mediated spine shrinkage 2 hours after corticosterone application, but not 40 min after?

We noticed that the 5 min pre-treatment data was missing from Fig. 6A. This has now been added back. For all other figure parts, 5 min baseline data is shown. Regarding the second question, the answer is yes. When ketamine was added 2h before CORT, the rescue was not visible by 40 min, but emerged by 2h.

Why did the authors use optogenetic stimulation in fig. 6 D,E but pharmacology in fig. 6F-H to inhibit JNK? dJNKIi1 was used nowhere else in this study. Why is it used here?

We could not use optogenetic stimulation for the longer time points because continuous live-cell imaging of these conditions would take 250 hours of confocal imaging (if everything went without glitches). Our Zeiss 880 is part of an imaging core and is in high demand. Usage per person is restricted to 4h per day. It would have been very expensive slow. The imaging experiments in figure 4 and 6 with Airyscan took about 200 hours. We therefore used pharmacology just to see if ketamine would at all rescue after a longer incubation. Moreover this makes the results more translational. Finally, we would like to point out that DJNKI-1 is the same peptide sequence as is encoded in the JBD of LOV2-JBD. As these experiments queried ketamine, which is a drug, a pharmacology type approach seemed like a reasonable solution.

Further, I assume that the axis labels “KET” and “dJNKi1” in fig. 6F-H in fact mean “KET+CORT” and “dJNKi1+CORT” (and the other way round), like in fig. 6 D,E?

Yes, this is correct. We have relabelled this figure and increased the size of the inset which shows timelines above the graphs.

With the missing baseline, the inappropriate spine head volume measurements and SEP-GluA2-normalization, I have doubts that the long-term effects of ketamine can be properly interpreted.

Baseline data has been added (Fig. 6A) as described above. We have added more methodological detail on how the spine volume was measured (respond to reviewer 2 point 1).

Finally, essential statistical analyses are missing or not applied properly. Statistical analysis is missing for figures 4 D,E; 5 C; 6 D,E. Moreover, a t-test cannot be used if more than two groups are present, as in fig. 5 F. Since this figure represents parts of a population, a chi square test would be more appropriate.

We have included new, additional statistical analysis to Fig. 4, 5 and 6. Including not only endpoint analysis but also entire timeline analysis (one-way ANOVA with post hoc Bonferroni testing). The details are described in the methods section. Adjusted p-values are highlighted on the figures. For Figure 5F, we have re-plotted the data so that it addresses only spine shrinkage which we think is clearer.

Costello DA, Herron CE (2004) The role of c-Jun N-terminal kinase in the A beta-mediated impairment of LTP and regulation of synaptic transmission in the hippocampus. Neuropharmacology 46:655-662.

Curran BP, Murray HJ, O'Connor JJ (2003) A role for c-Jun N-terminal kinase in the inhibition of long-term potentiation by interleukin-1beta and long-term depression in the rat dentate gyrus in vitro. Neuroscience 118:347-357.

Henson MA, Tucker CJ, Zhao M, Dudek SM (2017) Long-term depression-associated signaling is required for an in vitro model of NMDA receptor-dependent synapse pruning. Neurobiol Learn Mem 138:39-53.

Lee HK, Kameyama K, Huganir RL, Bear MF (1998) NMDA induces long-term synaptic depression and dephosphorylation of the GluR1 subunit of AMPA receptors in hippocampus. Neuron 21:1151-1162.

Lüscher C, Malenka RC (2012) NMDA receptor-dependent long-term potentiation and long-term depression (LTP/LTD). Cold Spring Harb Perspect Biol 4.

Sherrin T, Blank T, Todorovic C (2011) c-Jun N-terminal kinases in memory and synaptic plasticity. Rev Neurosci 22:403-410.

Sherrin T, Blank T, Hippel C, Rayner M, Davis RJ, Todorovic C (2010) Hippocampal c-Jun-N-terminal kinases serve as negative regulators of associative learning. J Neurosci 30:13348-13361.

Zhou Q, Homma KJ, Poo MM (2004) Shrinkage of dendritic spines associated with long-term depression of hippocampal synapses. Neuron 44:749-757.
