# Supplementary figures and images for: Optogenetic Control of Spine-Head JNK Reveals a Role in Dendritic Spine Regression
Source: eNeuro. 2020 Feb 12;7(1):ENEURO.0303-19.2019. doi: 10.1523/ENEURO.0303-19.2019 (PMC7053173; doi:10.1523/ENEURO.0303-19.2019)

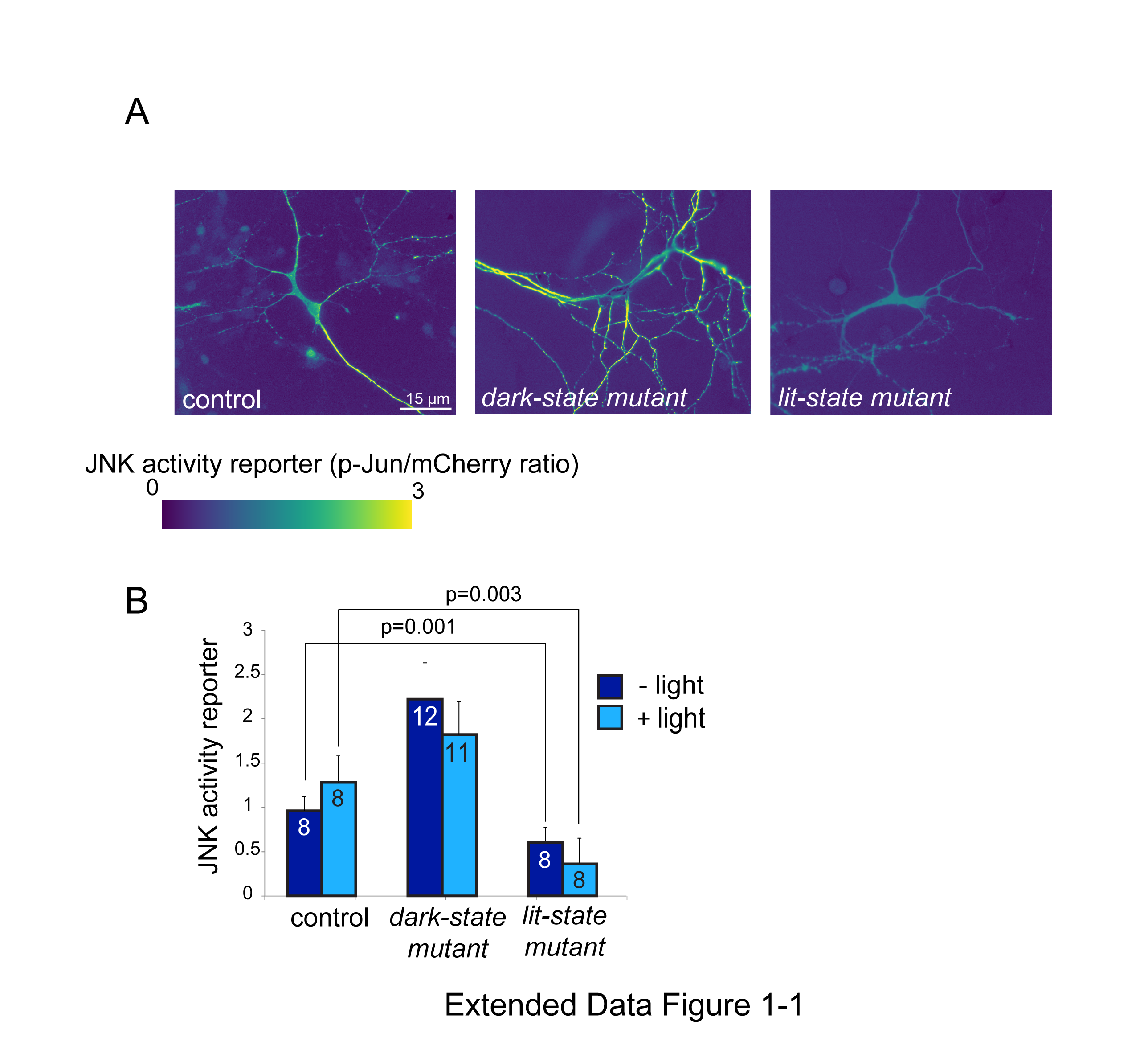

Supplement: Extended Data Figure 1-1 — Testing functionality of LOV2-JBD tools in neurons expressing mCherry-NES-Jun as a JNK activity reporter. A, Fluorescent micrographs from 16-d hippocampal neurons expressing the mCherry-NES-Jun reporter in the presence or absence (control) of the dark-state and lit-state mutants of LOV2-JBD as indicated. Micrographs show ratio images of phospho-Ser63-c-Jun (P-Jun)/mCherry-NES-Jun (mCherry) fluorescence. Scale bar = 15 μm. B, JNK activity (from multiple experiments as shown in A) is the ratio of phosphorylated c-Jun normalized to mCherry-NES-Jun reporter expression. The “lit-state” mutant of LOV2-JBD reduced JNK activity even without photostimulation. The “dark-state” mutant did not significantly alter JNK activity even in the presence of light. Mean data ± SEM are shown; p values are shown from repeated measures one-way ANOVA and are indicated above the histogram bars. Total number of cells analyzed from at least two experimental repeats are indicated on the bars. Download Figure 1-1, TIF file. [file sup_enu-eN-NWR-0303-19-s01.tif]

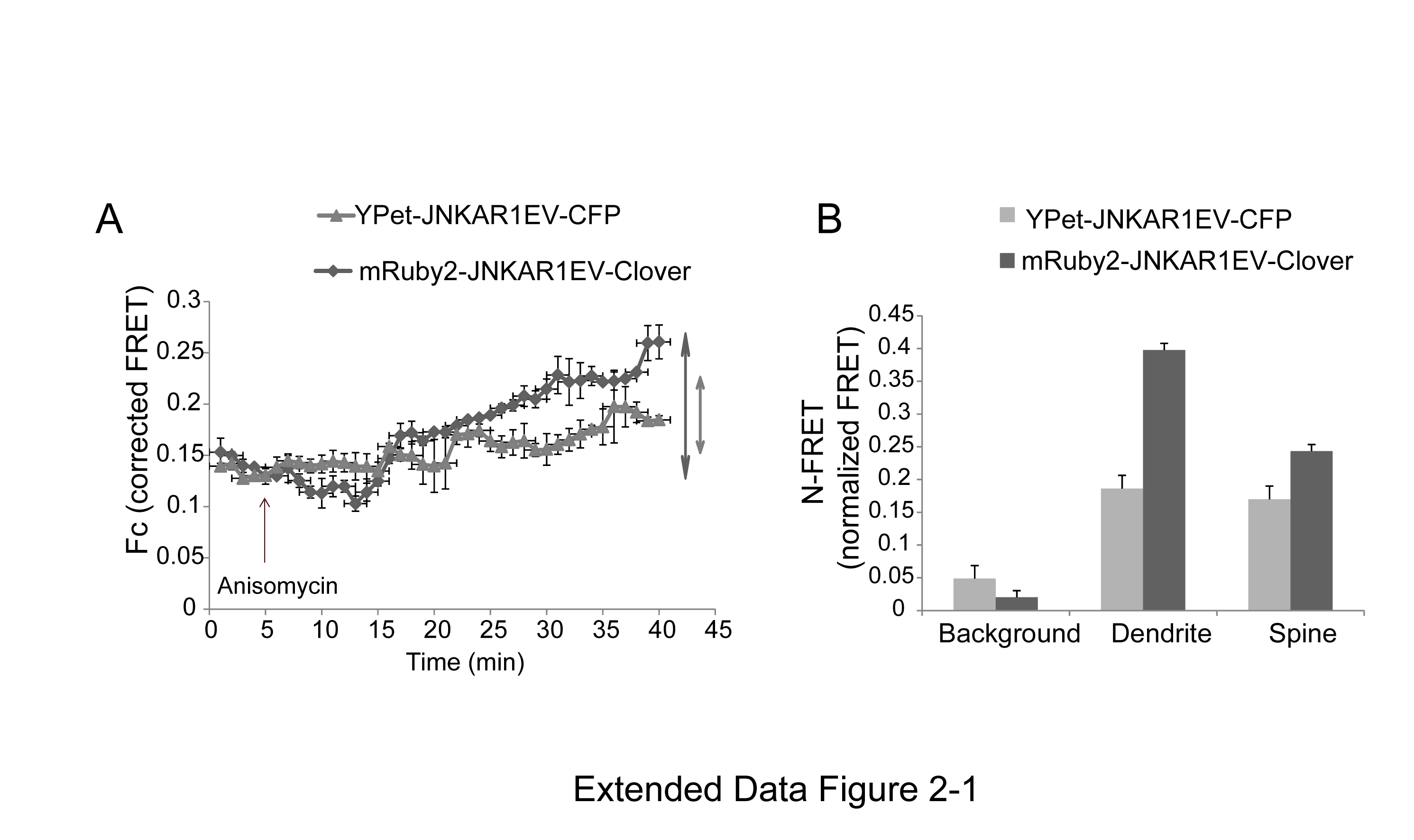

Supplement: Extended Data Figure 2-1 — mRuby2/Clover pairing improves JNK FRET reporter sensitivity. A, The sensitivity of FRET response was compared using the EYFP-JNKAR1EV-CFP FRET reporter and the newly generated mRuby2-JNKAR1EV-Clover reporter. Reporter activity was measured in 16-d hippocampal neurons treated with anisomycin (10 μM). B, N-FRET was measured from multiple cells expressing both reporters. mRuby2-JNKAR1EV-Clover provided improved dynamic range compared to EYFP-JNKAR1EV-CFP (dendrite, p = 0.000167). Measurements were from five regions per cell and four cells per experiment on two separate experiments. Mean data ± SEM are shown. Download Figure 2-1, TIF file. [file sup_enu-eN-NWR-0303-19-s02.tif]

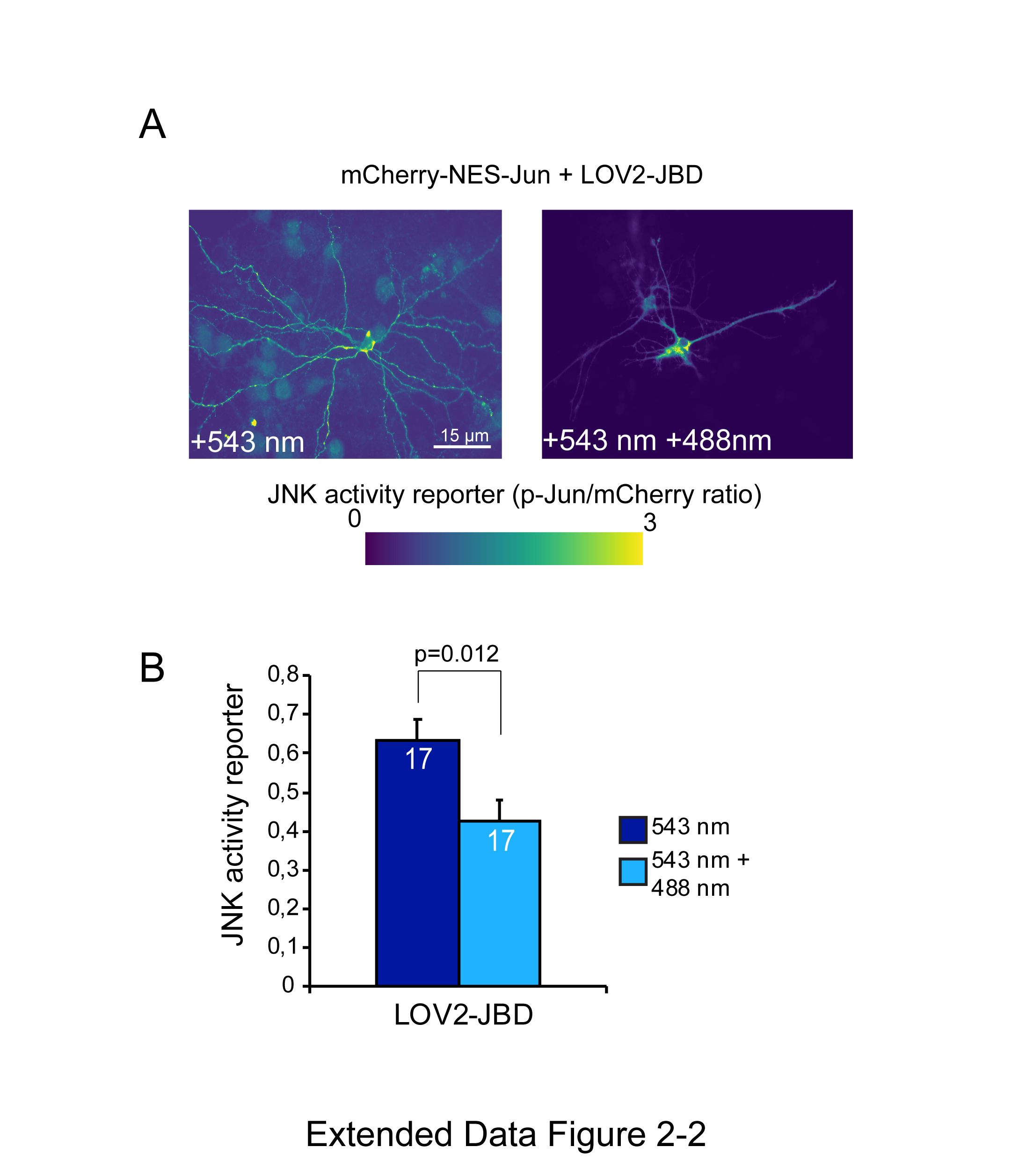

Supplement: Extended Data Figure 2-2 — The 488-nm irradiation in neurons expressing LOV2-JBD partially inhibits JNK. Fluorescent micrographs from 16-d hippocampal neurons expressing mCherry-NES-Jun+LOV2-JBD in the presence, or absence of 488-nm irradiation mimicking Clover/FRET channel excitation (0.4 mW). Micrographs show ratio images of phospho-Ser63-c-Jun (P-Jun)/mCherry-NES-Jun (mCherry) fluorescence. Scale bar = 15 μm. B, JNK activity (from multiple experiments as shown in A) is the ratio of phosphorylated c-Jun normalized to mCherry-NES-Jun reporter expression. Mean data ± SEM are shown, p value is obtained by Student’s t test. Download Figure 2-2, TIF file. [file sup_enu-eN-NWR-0303-19-s03.tif]

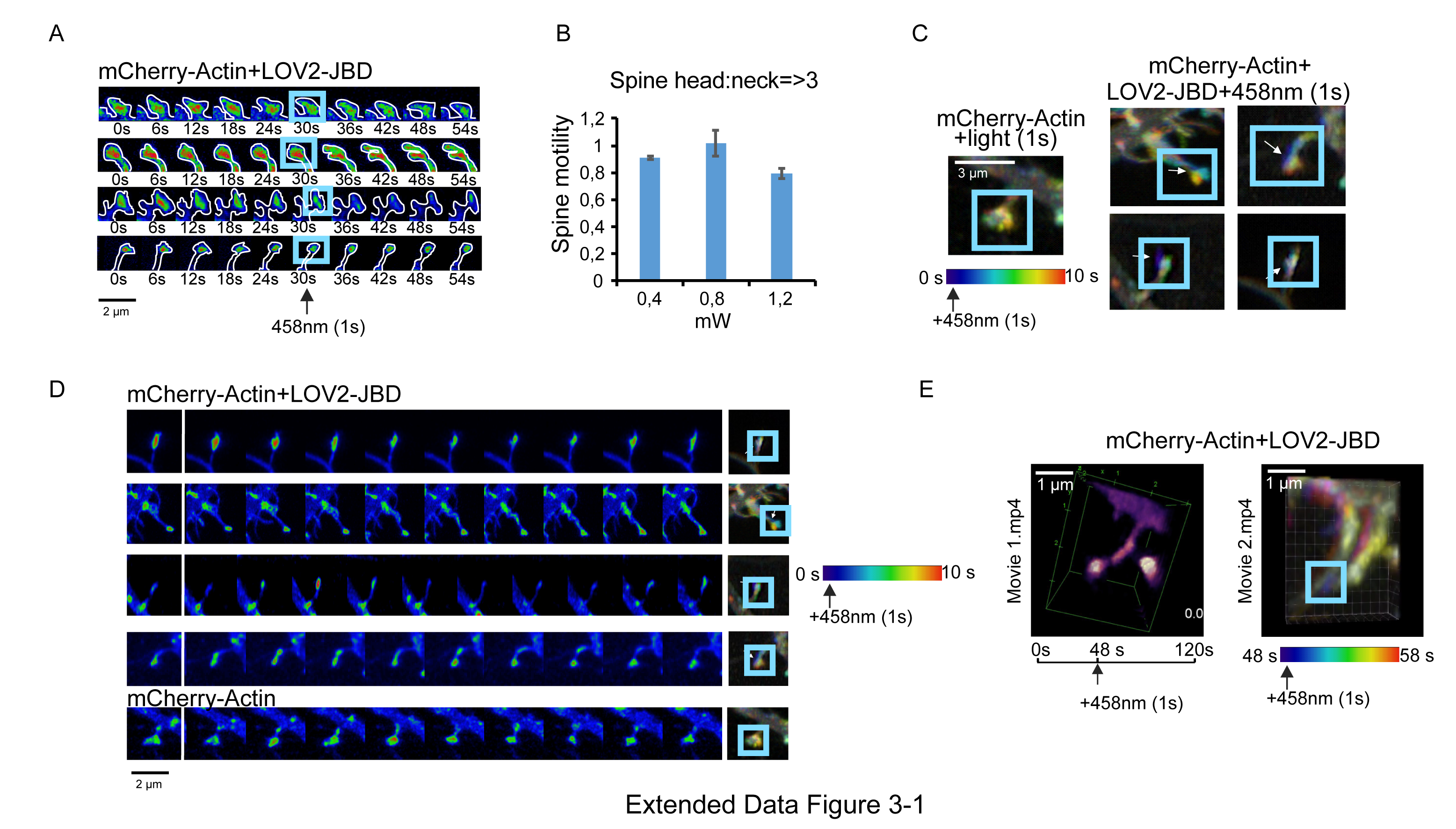

Supplement: Extended Data Figure 3-1 — Photostimulation of LOV2-JBD in dendritic spines rapidly immobilizes spine-head actin in the peripheral domain. A, Time-lapse sequences from 16-d hippocampal neurons expressing mCherry-actin and LOV2-JBD variants as indicated. Cells were stimulated for 1 s with 0.4 mW of 458-nm light. B, The effect on spine motility of LOV2-JBD photoactivation in mushroom spines (head diameter:neck length ratio >3) is shown. Spine motility is measured from arithmetic difference projection ratios (motility after light/motility before light). Optical stimulation of mushroom spines did not alter mCherry-actin motility in spines even when laser power was increased to compensate for larger spine-head volume. At least six spines from four experimental repeats were measured for each condition. Mean data ± SEM are shown. C, High-resolution maximum projections show temporally color-coded dendritic spines from 10-s time lapse from 3D recordings using Airyscan mode, at 1-s intervals. D, Footprints of time lapse sequences are shown for color-coded time projections from cells expressing mCherry-actin with or without LOV2-JBD for spines shown in C. E, Movies generated from 3D Airyscan recordings (1-s interval) of dendritic spines of a cell expressing mCherry-actin with LOV2-JBD as shown in Movie 1: time lapse movie 0-movie 120 s, blue circle depicts ROI of 458-nm illumination. Movie 2, volumetric temporal color coding of a dendritic spine generated from 48 to 58 s with 1-s 458-nm illumination at 48 s. Download Figure 3-1, TIF file. [file sup_enu-eN-NWR-0303-19-s04.tif]

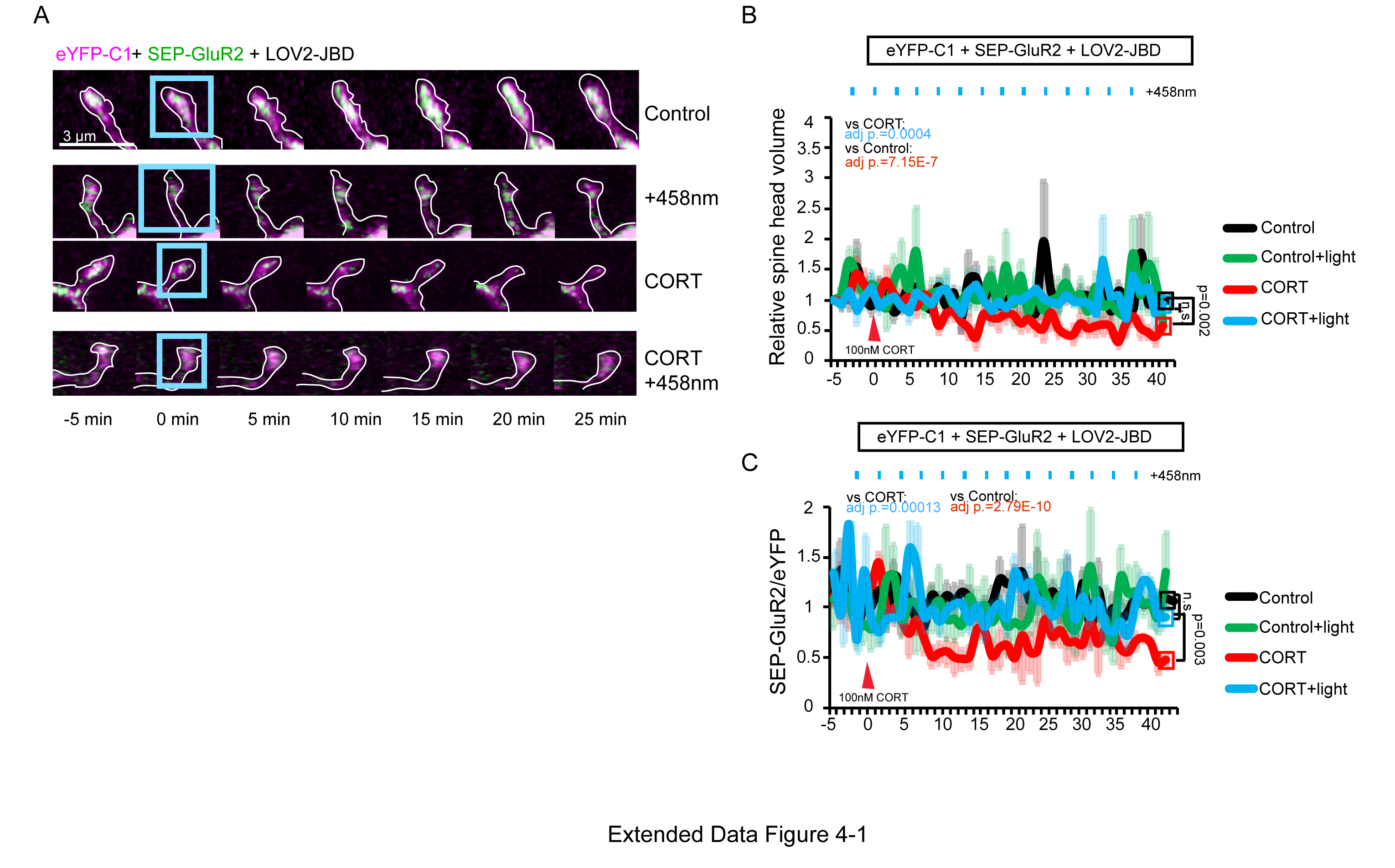

Supplement: Extended Data Figure 4-1 — Optogenetic inhibition of JNK recovers regressed spines and SEP-GluR2 induced by corticosterone. A, Time-lapse sequences from 16-d hippocampal neurons expressing eYFP-C1 (magenta), SEP-GluR2 (green), and LOV2-JBD. Cells were treated with 100 nM CORT at 0 min; 458-nm light pulses were applied every 3 min where indicated (+458 nm). B, Quantitative data show spine-head volume changes relative to baseline, calculated from six experiments. C. Quantitative data show plasma membrane SEP-GluR2 fluorescence normalized to YFP. Data are from six experiments as depicted in C. Adjusted p values (written on the graph) are from comparisons of full timelines from multiple experiments using repeated measures one-way ANOVA with Bonferroni correction. Endpoint averages are also shown. Download Figure 4-1, TIF file. [file sup_enu-eN-NWR-0303-19-s06.tif]
